# Using Polygenic Scores in Social Science Research: Unraveling Childlessness

**DOI:** 10.3389/fsoc.2019.00074

**Published:** 2019-11-22

**Authors:** Renske M. Verweij, Melinda C. Mills, Gert Stulp, Ilja M. Nolte, Nicola Barban, Felix C. Tropf, Douglas T. Carrell, Kenneth I. Aston, Krina T. Zondervan, Nilufer Rahmioglu, Marlene Dalgaard, Carina Skaarup, M. Geoffrey Hayes, Andrea Dunaif, Guang Guo, Harold Snieder

**Affiliations:** ^1^Department of Sociology and ICS, University of Groningen, Groningen, Netherlands; ^2^Department of Public Administration and Sociology, Erasmus University Rotterdam, Rotterdam, Netherlands; ^3^Department of Sociology and Nuffield College, University of Oxford, Oxford, United Kingdom; ^4^Department of Epidemiology, University of Groningen, University Medical Center Groningen, Groningen, Netherlands; ^5^Institute of Social and Economic Research, University of Essex, Essex, United Kingdom; ^6^École Nationale de la Statistique et de L'administration Économique (ENSAE), Paris, France; ^7^Center for Research in Economics and Statistics (CREST), Paris, France; ^8^Department of Surgery, University of Utah, Salt Lake City, UT, United States; ^9^Wellcome Centre for Human Genetics, University of Oxford, Oxford, United Kingdom; ^10^Department of Bio and Health Informatics, Technical University of Denmark, Lyngby, Denmark; ^11^Department of Growth and Reproduction, Rigshospitalet, Copenhagen, Denmark; ^12^Division of Endocrinology, Metabolism, and Molecular Medicine, Department of Medicine, Northwestern University Feinberg School of Medicine, Chicago, IL, United States; ^13^Center for Genetic Medicine, Northwestern University Feinberg School of Medicine, Chicago, IL, United States; ^14^Department of Anthropology, Northwestern University, Evanston, IL, United States; ^15^Department of Endocrinology, Diabetes and Bone Disease, Icahn School of Medicine at Mount Sinai, New York, NY, United States; ^16^Department of Sociology, University of North Carolina at Chapel Hill, Chapel Hill, NC, United States

**Keywords:** fertility, childlessness, polygenic risk scores, sociogenomics, infertility

## Abstract

Biological, genetic, and socio-demographic factors are all important in explaining reproductive behavior, yet these factors are typically studied in isolation. In this study, we explore an innovative sociogenomic approach, which entails including key socio-demographic (marriage, education, occupation, religion, cohort) and genetic factors related to both behavioral [age at first birth (AFB), number of children ever born (NEB)] and biological fecundity-related outcomes (endometriosis, age at menopause and menarche, polycystic ovary syndrome, azoospermia, testicular dysgenesis syndrome) to explain childlessness. We examine the association of all sets of factors with childlessness as well as the interplay between them. We derive polygenic scores (PGS) from recent genome-wide association studies (GWAS) and apply these in the Health and Retirement Study (*N* = 10,686) and Wisconsin Longitudinal Study (*N* = 8,284). Both socio-demographic and genetic factors were associated with childlessness. Whilst socio-demographic factors explain 19–46% in childlessness, the current PGS explains <1% of the variance, and only PGSs from large GWASs are related to childlessness. Our findings also indicate that genetic and socio-demographic factors are not independent, with PGSs for AFB and NEB related to education and age at marriage. The explained variance by polygenic scores on childlessness is limited since it is largely a behavioral trait, with genetic explanations expected to increase somewhat in the future with better-powered GWASs. As genotyping of individuals in social science surveys becomes more prevalent, the method described in this study can be applied to other outcomes.

## Introduction

Childlessness has increased in many Western countries, from 10% in the 1970s to currently 15% in the US (Frejka, 2017). Childlessness can have far reaching consequences, including changing the age composition of the population and lower well-being among the involuntary childless (Sleebos, 2003; Hansen et al., 2009).

Three parallel strands of research have examined reproductive behavior. Firstly, the social sciences examined socio-demographic factors such as educational attainment, occupational behavior, religiosity, marital status, and birth cohort (Balbo et al., 2013). Secondly, medical research has focused on fecundity, infertility or the biological ability to conceive such as sperm defects and ovulatory, cervical, fallopian tube and uterine problems (Blundell, 2007). Thirdly, a growing body of research focuses on the genetics of fertility outcomes, such as age at first birth (AFB), number of children born (NEB) and childlessness, with twin and family studies showing that genetics may explain up to 50% of the variation in AFB, NEB and childlessness (Mills and Tropf, 2015; Tropf et al., 2015; Verweij et al., 2017). Recent Genome-wide Association Study (GWAS) discoveries have isolated genetic markers for reproductive behavior such as the timing and number of children (Barban et al., 2016) and more biologically based infertility traits related to sperm defects or the timing of menopause (Painter et al., 2011; Day et al., 2015), allowing us for the first time to include an individual's genetic propensity as predictors in our statistical models.

Until now, these three strands of research have existed in isolation (Mills and Tropf, 2015), largely due to absence of data, training or realization of the importance of adopting a combined sociogenomic approach. The result is a lack of understanding of the relationship between biological, genetic and socio-demographic factors in association with childlessness. We also do not know whether estimates based solely on socio-demographic factors are biased due to their correlation with an individual's genetic propensity (Tropf and Mandemakers, 2017) or if genetic propensities interact with socio-demographic factors to be more influential in particular groups. Using known socio-demographic measures and results from recent GWAS discoveries, we apply a novel design in which polygenic scores (PGSs) are created for a variety of behavioral and infertility-related reproductive outcomes. Due to the novelty of our design, we use two independent datasets to replicate our results, namely the two US-based Health and Retirement Study (HRS, *N* = 10,686) and the Wisconsin Longitudinal Study (WLS, *N* = 8,284). Both include individuals born between 1920 and 1960, where childlessness rose from 6% among women born in 1935 to 16% around 1950 (Human Fertility Database, 2017) (see Figure SM1). We first introduce our conceptual model, followed by an explanation of the data and methods, main results and implications for future research related to childlessness and beyond.

### Conceptual Model and Expectations

**Figure 1:**
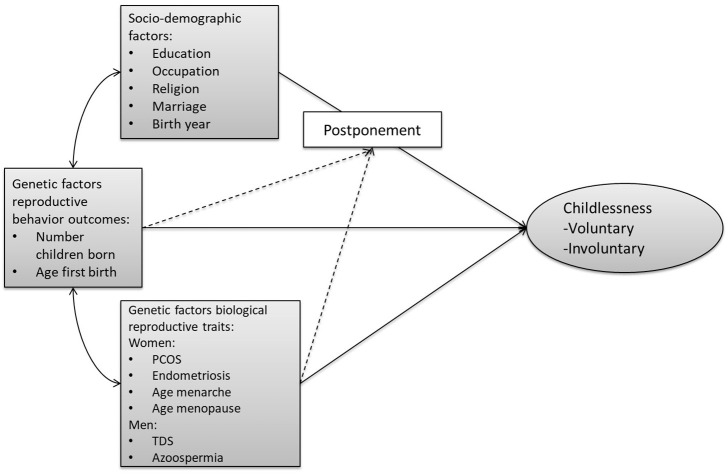
Conceptual model on the pathways from the three sets of factors leading to (both voluntary and involuntary) childlessness. The arrows from the three sets of factors (socio-demographic, genetic reproductive behavior, genetic biological reproductive traits) to childlessness represent the expected main effects. The dashed lines represent the expected interaction between PGSs and socio-demographic factors. The double-sided arrows represent the expected correlations and the single-headed arrows the flow of causality. PCOS, polycystic ovary syndrome; TDS, testicular dysgenesis syndrome.

Figure 1 provides an overview of our conceptual model. Unfortunately, we are not able to distinguish between voluntary and involuntary childlessness, so all childless individuals are combined into one group. We first assess the relationship of (1) socio-demographic factors, (2) genetic factors related to biological reproductive traits (e.g., menarche, sperm defects), and (3) genetic factors related to reproductive behavior (timing, number of children) with childlessness. We acknowledge that this trichotomy is not entirely mutually exclusive, given that some of the socio-demographic factors have been shown to have at least a partial genetic basis (e.g., educational attainment, religiosity). Furthermore, pathways through which the PGSs relate to childlessness might operate via the socio-environment. However, we use this clustering since it reflects the divisions and representations in the literature.

The central socio-demographic factors that we study are education, work, religion, marriage, and birth year (Balbo et al., 2013). Previous studies showed that higher education and full-time work are associated with higher chances of childlessness among women, but not among men (Keizer et al., 2008; Balbo et al., 2013; Tropf and Mandemakers, 2017). More religious individuals are less likely to remain childless (Frejka and Westoff, 2008). Furthermore, in the US most childbearing happened within marriage and therefore men and women who got married younger are less likely to remain childless (Ventura and Bachrach, 2000). Birth year is also important, as childlessness is more prevalent among individuals born in 1960 (15%) than in 1940 (8%) (Human Fertility Database, 2019).

We include PGS for biological reproductive traits on which GWAS studies have been conducted. For women, ovulatory, cervical, fallopian tube, and uterine problems are most likely to cause infertility (Blundell, 2007) and therefore we include genetic scores for polycystic ovary syndrome (PCOS) (Hayes et al., 2015) (which mainly cause ovulatory problems), endometriosis (Painter et al., 2011) (which influences the ovaries and fallopian tubes), age at menarche (Day et al., 2017), and age at menopause (Day et al., 2015) (which determine women's reproductive life span). For men, sperm defects are the most likely cause for infertility, therefore we include PGSs for azoospermia (a condition in which the semen contains no sperm) and oligiozoospermia (low sperm count) (Aston and Carrell, 2009) and testicular dysgenesis syndrome (TDS) (Dalgaard et al., 2012).

We also include genetic scores for reproductive behavior, namely the age at first birth (AFB) and number of children ever born (NEB) (Barban et al., 2016). These genetic scores likely capture both social pathways leading to childlessness, such as desires for a certain family size and educational attainment, but also biological pathways, such as sperm defects or ovulatory functioning.

We further study the interaction between these socio-demographic and genetic factors. We hypothesize a birth cohort by genetic score interaction based on studies that demonstrate that there are differences in the relationship between genes and reproductive outcomes over time (Kohler et al., 2002; Briley et al., 2015; Tropf et al., 2015, 2017). We also hypothesize that genetic factors are more important for men and women who get married, and thus start attempts to have children, at higher ages, because the biological ability to conceive decreases with age, especially for women (Menken et al., 1986).

Finally, we assess genetic (G-G) and gene by socio-demographic (G-E) correlations and mediation. We expect a shared genetic basis reflected in correlations between the genetic scores for the biological reproductive traits and genetic scores for reproductive behavior (G-G correlation) (Barban et al., 2016), as well as between education and age at marriage with the genetic scores for age at first birth and number of children born(G-E correlation) (Briley et al., 2017). We examine if the effects of genetic scores for reproductive behavior are mediated by socio-demographic factors and genetic scores for the biological reproductive traits. Due to biological differences between men and women, we likewise examine sex differences (Verweij et al., 2017), and also explore differences by ethnic groups (Ware et al., 2017).

## Methods and Materials

### Data, Genotyping, and Samples

We use two broadly comparable datasets from the US, namely the Health and Retirement Survey and the Wisconsin Longitudinal Study.

#### Health and Retirement Survey (HRS)

The HRS is a nationally representative sample of men and women born between ~1920 and 1960 living in the US. This survey started in 1992 with a sample of men and women aged 51–61 and their partners, interviewed every 2 years. Extra cohorts have been added to create a representative sample of Americans over 50 years of age (Sonnega et al., 2014), resulting in over 27,000 respondents in 2010 (Health Retirement Study, 2017).

In 2006, the HRS started genotyping respondents, with data from 15,445 individuals currently available. In 2006, half of the entire living sample was asked to provide saliva samples for genotyping, of which 83% gave saliva, in 2008 the other half of the sample was asked of which 84% gave saliva and in 2010 half of the newly added HRS sample was asked of which 80% gave saliva (Weir, 2013; HRS, 2017). Genotyping of the 2006–2008 samples was done by the Illumina HumanOmni-2.5 Quad BeadChip, with coverage of ~2.5 million single nucleotide polymorphisms (SNPs). Genotyping of the 2010 sample was done with the Illumina HumanOmni2.5-8v1 BeadChip (HRS, 2017). This chip covers common, rare, and exonic SNP content from the 1,000 Genomes Project. Based on self-reported ethnicity we selected only the white non-Hispanic sample (*N* = 10,686), and we conducted separate analyses on the black non-Hispanic sample (*N* = 2,433) (we removed the Hispanic sample and people with other ethnicities).

#### Wisconsin Longitudinal Study (WLS)

The WLS is a random sample of one third of all men and women who graduated from Wisconsin high schools in 1957 (*N* = 10,317 graduates), and one of their siblings (*N* = 8,734 siblings). It is broadly representative of white, non-Hispanic Americans who at least finished high school (Herd et al., 2014). Respondents filled in questionnaires across six waves (1957, 1964, 1975, 1993, 2004, 2011).

Between 2007 and 2011, 9,012 of the WLS respondents were genotyped. From the total of 10,317 graduates in the sample 5,967 gave DNA and consent, 2,394 refused, 1,657 already deceased and 298 were not found (68.9% of the living samples gave DNA) (WLS, 2011). From the siblings, 3,440 gave DNA and consent, 1,415 refused, 1,514 already deceased, and 2,412 were not found (42.7% living respondents gave DNA). Genotyping was done using the Illumina HumanOmniExpress-24-v1-1 BeadChip that includes 713,014 SNPs (Herd, 2016). The SNPs on this chip were optimized to tag content from all three HapMap phases, which combine both rare as common genetic variation (Altshuler et al., 2010). We select only those individuals who provided information about their number of children after they finished their reproductive period (age 45 for women or 50 for men), resulting in 8,284 individuals.

In both samples, there were no individuals with missing call rates of over 2%. In the HRS we removed all genetically related individuals, for this we used the kinship coefficient table that is provided by HRS which is based on SNP similarity between individuals, removing individuals with similarity >0.125 (Weir, 2013). In the WLS we used multilevel models to deal with related individuals. The reason is that the inclusion of related individuals could result in an inflated significance of the SNP effects. We did not impute genetic data but only used raw genotyped information. Further information on quality control is provided in the quality control reports for both samples (Weir, 2013; Herd, 2016).

### Measurements

*Childlessness* is measured from a direct question regarding the number of biological children after reaching the end of their reproductive period.

*Birth year of respondent* is the birthdate reported in the first non-missing wave and is standardized [(value-mean)/SD] for ease of comparison.

*Years of education* is the number of years of education and is calculated based of the highest degree (asked at least once after the age of 30) and is also standardized.

*Occupational field* is measured in the HRS by job asking respondents about their job previous to their current occupation distinguishing between “professionals,” “managers,” “clerks,” “sales,” “mechanics/production,” “services,” “operators,” “farming,” and “army.” In the WLS, it is measured by the first job they had after completing the highest level of schooling, distinguishing between “professional/technical,” “administrators/managers,” “sales,” “clerks,” “manufacturing/construction,” “service,” “farming,” and “no first job.” In both datasets clerks were used as reference groups.

*Age at first marriage* is measured in both datasets using information from the total number of marriages at each wave, using the answer at the last interview. It is dichotomized into never and ever married and for those who had been ever married, the age of their first marriage, categorized into “before 21,” “21–25,” “26–30,” “31–35,” “36–40,” and “older than 41” years. In this time period most childbearing occurred within marriage, from 98% (1940–1960), to 94% in 1970, 90% in 1980, and 80% in 1990 (Ventura and Bachrach, 2000).

*Religion* in the HRS respondents were asked their religious preference at each wave: “Protestant,” “Roman Catholic,” “Jewish,” “something else,” or “non-religious.” The answer from the first wave with non-missing information was used. In several waves of the WLS, respondents were asked about their current religious preference and could choose between 76 religions, which we collapsed into Roman Catholic, Protestant, other, and not religious. The answer from the first wave with non-missing information was used.

*Ethnicity* in the WLS we used self-reported ethnicity, removing non-white respondents. In the HRS, respondents were asked: “Do you consider yourself primarily: ‘White or Caucasian,’ ‘Black or African American,’ ‘American Indian,’ or ‘Asian’?” Respondents were also asked if they identified as Hispanic, and the Hispanic respondents were removed from the sample. Since the HRS oversampled black individuals, we were able to create a white and black sample.

#### GWASs Used to Create PGSs

We used single nucleotide polymorphisms (SNPs) and their summary statistics for *NEB* and *AFB*, which were obtained from a recent GWAS that used 251,151 European ancestry individuals for AFB and 343,072 individuals for NEB (Barban et al., 2016). For *endometriosis* a GWAS of 3,194 surgically confirmed endometriosis cases (of which 1,364 moderate-severe) and 7,060 controls from Australia and the United Kingdom was used (Painter et al., 2011). The *PCOS* GWAS consisted of 984 PCOS cases and 2,946 controls, all of European ancestry (Hayes et al., 2015). For *age at menarche*, defined by age at first menstrual period, we used the GWAS of 329,345 women from European ancestry (Day et al., 2017). For *age at menopause*, defined as the age at which a woman had her last menstrual period, data from the GWAS that included 69,360 women of European ancestry were used (Day et al., 2015). *Azoospermia and oligozoospremia* data stem from a GWAS of 80 controls, 52 oligozoospermia cases and 40 azoospermia cases, including white individuals primarily of northern European descent (Aston and Carrell, 2009). For *TDS* results were used of a GWAS, that included 488 cases and 439 controls from Denmark (Dalgaard et al., 2012). Of these cases 107 were infertile with sperm count below 15 million per milliliter (ml) in the semen and testis volume below 15 ml, 212 with testicular germ cell tumors (TGCC), 138 with cryptorchidism, and 31 with hypospadias.

#### Creation of PGS

To examine the impact of the genetic factors on childlessness we created separate PGSs, using GWAS summary statistics by calculating the sum of all risk alleles, weighted by their reported effect sizes. A PGS thus can be seen as the summary measure of the genetic propensity for a trait (Wray et al., 2007). PGSs were created with the PRSice tool (Euesden et al., 2015) in PLINK. We use linkage disequilibrium (LD) clumping, for which an *r*^2^ threshold of 0.1 and a distance threshold of 250 kb were used, indicating that if two SNPs have a squared correlation of 0.1 or greater, or a distance of 250 kb or smaller, only one of the two SNPs is included in the PGS. We included only genotyped SNPs, as opposed to also using imputed SNPs, because including imputed SNPs generally does not increase predictive power (Ware et al., 2017). Different PGSs were created depending on *P*-value cutoffs, from using only genome wide significant SNPs (*P* ≤ 5 × 10^−8^) to including all genotyped SNPs (*P* ≤ 1) (see Figures SM2–SM5). In our main analyses we included the PGSs that include all genotyped SNPs, since these scores generally had the highest explained variance in our samples. See Table SM6 for the number of SNPs included in each PGS. A requirement for using PGSs is that the GWAS sample is independent of the sample in which the PGS is applied (Wray et al., 2013). The HRS sample was included in the GWASs for NEB, AFB and age at menopause. For that reason for NEB and AFB we received GWAS summary statistics excluding the HRS sample from the authors of the GWAS. For age at menopause we used the MetaSubtract package in R (Nolte, 2017; Nolte et al., 2017) to subtract the HRS GWAS results from the Meta GWAS results on age at menopause.

#### Principal Components

We had to control for population stratification, which is the case if certain SNPs are more common in certain regional or ancestral populations, which would result in a false effect of the PGSs on the outcome if the outcome shows regional/ancestral variation (Price et al., 2006). We therefore include the first 20 principal components (PCs) from the genomic relationship matrix for all individuals using SNPs, which is generally sufficient to capture regional genetic variation.

For both the HRS and the WLS samples, the PCs are provided through dbGaP (Weir, 2013; Herd, 2016). The principal component analysis is performed after pruning based on LD, including only SNPs with missing call rate <2% (WLS) or <5% (HRS) and minor allele frequency >5%. A number of SNPs on certain regions [2q21 (LCT), HLA, 8p23, and 17q21.31 regions] are removed to avoid PCs to be largely influenced by small sets of SNPs. For HRS using the first seven PCs should be sufficient and for the WLS using the first six PCs should be sufficient (Weir, 2013; Herd, 2016). We use the conservative approach and include all 20 PCs in our analyses.

### Statistical Analyses

We apply logistic regression models, adding variables over four steps: (model 1) PGSs for behavioral genetic reproductive outcomes (AFB, NEB) with the first 20 PCs; (model 2) PGSs for biological fecundity-related genetic outcomes (including PCs); (model 3) socio-demographic factors; and, (model 4) all variables. To compare the explanatory power of the genetic and socio-demographic factors, we compare odds ratios (with both PGS and continuous variables standardized) and use adjusted McFadden's pseudo *R*^2^ [which is calculated as 1-(log(Lc)-k/log(Lnull)) where Lc is the likelihood value of the complete model and Lnull is the likelihood of the null model without covariates and k is the number of coefficients]. Since siblings are included in the WLS, we run multilevel models on respondents nested within households to adjust for non-independence (Snijders and Bosker, 2012).

The interaction between the genetic propensities and postponement of childbearing were examined by including all PGSs by age at first marriage interactions. To test whether genetic influences on fertility became stronger in more recent birth cohorts, we included a PGS (AFB and NEB) by birth year interaction. To properly control for confounding in gene^*^socio-demographic interaction models, Keller (2014) argues that interactions between confounders and genes as well as confounders and socio-demographics should be included. For that reason, we include interactions with the first five PCs and with education, birth year and religion.

To assess whether the effect of AFB and NEB PGSs is mediated/confounded by education, marriage or reproduction-related biological traits on the effect of on childlessness, simply comparing coefficients across models with and without confounding factors is not feasible, because unobserved heterogeneity differs between logistic regression models (Mood, 2010). We therefore apply the Karlson-Holm-Breen (KHB) method to equalize the scale of the log-odds across models (Karlson et al., 2012). With these models we can assess the percentage of confounding due to the PGSs and socio-demographic factors, after we control for the first 20 genetic PCs. We furthermore examine correlations between the AFB and NEB PGSs and education and marriage using Pearson correlation coefficients, as well as the association between AFB and NEB PGSs with the biological traits PGSs (while controlling for the first 20 principal components). In addition, we assess the LD-score genetic correlations between the reproductive behavior and the biological traits PGSs to assess the extent to which genes are shared between the traits (Bulik-sullivan et al., 2015). This method only requires summary statistics of GWAS results to estimate the genetic correlation between different traits and is not biased by sample overlap. We performed the LD-score correlation analyses in LD Hub (Zheng et al., 2017).

To estimate sex differences, we used the HRS and WLS samples on both men and women, for which we apply multilevel models (siblings in the WLS and partners in the HRS sample) including interactions with sex. To examine differences by ethnic groups, we run separate analysis on the black HRS sample, as well as analyses that includes both the black and the white sample, in which we include interactions with ethnicity. In our analyses we use self-reported ethnicity. We examined the overlap between self-reported ethnicity and values on the PCs. A small part of the individuals indicated to be white but differed from other white respondents on their PC values. As a robustness check we removed these individuals from the sample, either based on visual inspection or Mahalanobis distance, and results remained largely comparable.

## Results

### Descriptives

Descriptive statistics of the samples can be found in Table SM1. In the HRS 10.8% of women and 12.5% of men remained childless, whereas for the WLS this was 6.6% and 6.3% (these estimates are in line with the levels of childlessness in the US in these periods, see Figure SM1). In the HRS, around half of the respondents completed high school or less and around 20% of women finished college or above compared to 29% of men. In the WLS, around 58% of women finished high school only compared to 49% of men. Finishing college or more was 25 and 35% for women and men, respectively. Only a very small percentage in both samples never got married (between 3 and 4%). WLS respondents on average had younger ages at marriage than the HRS respondents.

### PGSs for Reproductive Behavior and Not Biological Traits Relate to Childlessness

**Figure 2:**
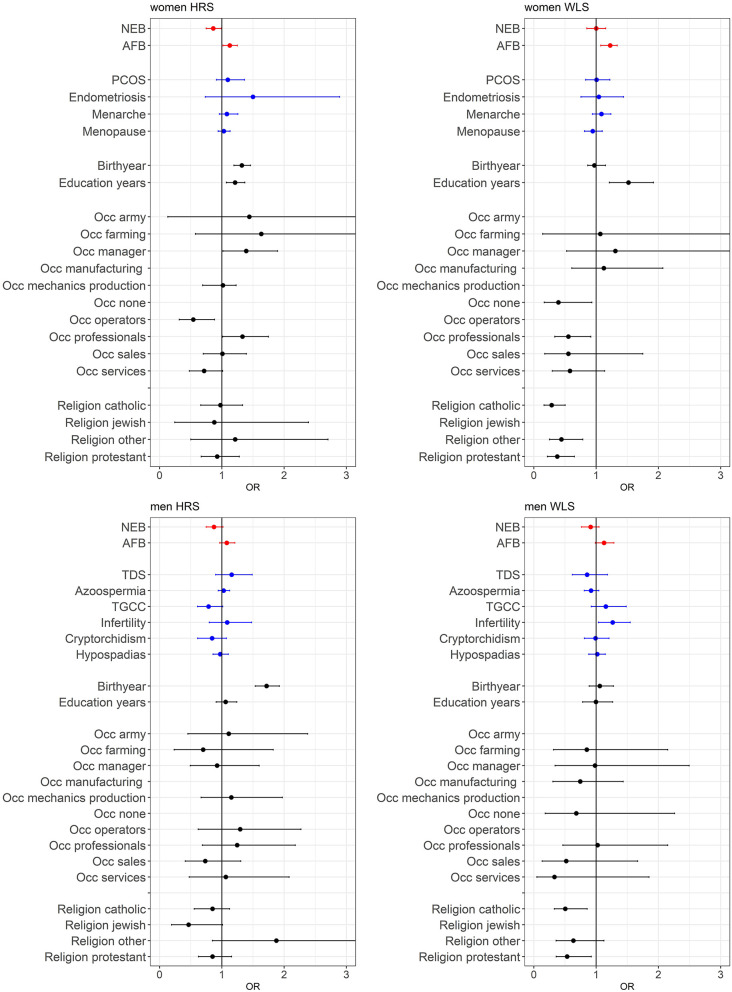
Effects of polygenic risk scores and socio-demographic factors on remaining childless, OR with 95% confidence intervals presented. The effect of marriage is displayed separately in Figure 3, since these effects are very large. See Tables SM2–SM5 for the complete regression tables. Estimates are based on three separate models; model 1 with PGSs for AFB and NEB and 20 PC's (red estimates), model 2 with PGS for biological reproductive traits and 20 PC's (blue estimates), and model 3 with socio-demographics (black estimates).

A main finding is that the PGSs for reproductive behavior (AFB and NEB) are to a small extent related to childlessness, while for the PGSs related to biological traits (PCOS, Endometriosis, Menarche and Menopause for women and TDS, Azoospermia, TGCC, Infertility, Cryptorchidism, and Hypospadias for men) we did not find an association with childlessness. PGSs favoring higher NEB were associated with smaller chances of remaining childless among both sexes, but only significantly in the HRS (Figure 2 and model 1 of Tables SM2–SM5). PGSs for later AFB were associated with higher chances of childlessness, especially among women (Figure 2 and model 1 of Tables SM2–SM5). The correlation between the AFB and NEB PGSs is relatively high, −0.17, −0.18, −0.29, and −0.24 in the female HRS, male HRS, female WLS, and male WLS samples, respectively (see Table 1). In Figures SM2–SM5 it is shown that when included in the model separately AFB is significant in all four samples and NEB in both male and female samples of the HRS. However, if we include all socio-demographic variables in our models, the effect sizes of the genetic scores decrease or become insignificant (see model 4 in Tables SM2–SM5, we elaborate on this further in the section on gene-socio-demographic correlations).

**Table 1:** Correlations between genetic and socio-demographic factors, and genetic correlations, based on PGSs in the HRS and WLS samples and based on LD-score regressions (LDSC).

	**HRS women**			**WLS women**		**LDSC**			
	***r***	**SE**	***p***	***r***	**SE**	***p***	***r***	**SE**	***p***
**AFB PGS**									
Education years	0.143	0.016	0.000	0.122	0.0161	0.000	0.72	0.021	0.000
Age marriage	0.005	0.002	0.007	0.035	0.0038	0.000			
Ever married	−0.028	0.065	0.655	−0.167	0.0795	0.050			
NEB PGS	−0.17	0.009	0.000	−0.29	0.0154	0.000	−0.66	0.033	0.000
PCOS PGS	0.067	0.024	0.006	0.011	0.0222	0.628	0.26	0.086	0.003
Endometriosis PGS	0.027	0.081	0.893	−0.084	0.0370	0.024	−0.09	0.076	0.218
Menarche PGS	0.101	0.016	0.000	0.089	0.0155	0.000	0.25	0.047	0.000
Menopause PGS	0.035	0.012	0.004	0.059	0.0172	0.001	0.20	0.052	0.000
	**HRS men**			**WLS men**					
Education years	0.143	0.016	0.000	0.088	0.014	0.000			
Age marriage	0.005	0.002	0.007	0.019	0.003	0.000			
Ever married	−0.028	0.065	0.665	−0.132	0.077	0.085			
NEB PGS	−0.18	0.011	0.000	−0.24	0.014	0.000			
TDS PGS	0.019	0.013	0.117	0.016	0.015	0.283			
Azoospermia PGS	−0.021	0.015	0.161	−0.008	0.015	0.601			
TGCC PGS	0.056	0.020	0.004	0.032	0.016	0.044			
Infertility PGS	−0.018	0.029	0.542	−0.007	0.018	0.690			
Cryptorchidism PGS	0.032	0.021	0.110	−0.008	0.016	0.603			
Hypospadias PGS	0.006	0.016	0.635	0.009	0.015	0.573			
**NEB PGS**									
	**HRS women**			**WLS women**			**LDSC**		
	***r***	**SE**	***p***	***r***	**SE**	***p***	***r***	**SE**	***p***
Education years	−0.046	0.013	0.000	−0.034	0.016	0.033	−0.263	0.031	0.000
Age marriage	0.000	0.001	0.912	−0.017	0.004	0.000			
Ever married	0.071	0.052	0.168	0.006	0.077	0.940			
PCOS PGS	−0.032	0.017	0.064	−0.028	0.021	0.171	−0.29	0.103	0.006
Endometriosis PGS	0.06	0.059	0.276	−0.047	0.034	0.173	−0.04	0.093	0.705
Menarche PGS	−0.007	0.012	0.599	−0.014	0.014	0.323	−0.01	0.054	0.884
Menopause PGS	0.012	0.009	0.213	0.00	0.016	0.966	−0.15	0.065	0.024
	**HRS men**			**WLS men**					
Education years	−0.046	0.013	0.000	−0.006	0.013	0.681			
Age marriage	0.000	0.001	0.912	−0.009	0.003	0.008			
Ever married	0.071	0.052	0.168	0.194	0.073	0.008			
TDS PGS	−0.009	0.009	0.346	0.007	0.014	0.614			
Azoospermia PGS	−0.006	0.011	0.539	0.002	0.014	0.887			
TGCC PGS	−0.025	0.015	0.113	0.00	0.015	0.997			
Infertility PGS	−0.004	0.022	0.912	0.031	0.016	0.052			
Cryptorchidism PGS	0.006	0.016	0.709	0.004	0.015	0.812			
Hypospadias PGS	−0.008	0.012	0.625	0.014	0.014	0.319			

*r, correlation; SE, standard error; p, p-value; PGS, polygenic risk score; NEB, number of children ever born; AFB, age at first birth; PCOS, polycystic ovary syndrome; TDS, testicular dysgenesis syndrome; TGCC, testicular germ cell tumors. All genetic correlations based on PGSs are controlled for the first 20 PCs. +LD score genetic correlation between AFB and NEB and the male biological reproductive traits are not calculated, because the GWASs on which these are based do not meet the sample size criteria required for LD score correlation analysis*.

For the PGSs related to biological fecundity we find only small and mixed findings (Figure 2 and model 2 of Tables SM2–SM5). For men, PGSs related to infertility due to low sperm count are related to lower levels of male childlessness, which we find only in the WLS sample. We do not find any associations between the PGSs related to biological fecundity traits that replicate across samples. This smaller and insignificant associations with the biological fecundity PGSs is likely attributed to the lower-powered GWASs they are based on (see Table SM6). The relationship between the PGSs and childlessness using different *p*-value cutoffs are graphically displayed in Figures SM2–SM5, showing that in most cases the *p*-value cutoff of 1 resulted in the highest odds ratios and smallest confidence intervals.

### Effect of Socio-Demographic Factors as Expected

**Figure 3:**
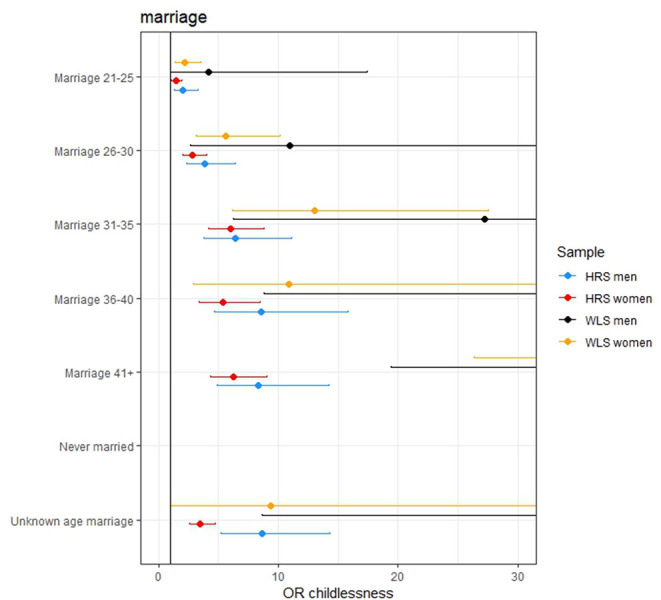
The effect of age at marriage on remaining childless. The effect of never being married is excluded from the figures since these effects are very large (OR 120.268, 471.383, 229.776, and 1,896 in the women HRS, women WLS, men HRS, and men WLS, respectively). The effects of the later ages at marriage are not completely displayed in some of the figures because these effects are large.

Individuals from more recent birth cohorts, those who married older or did not marry and were not religious (in the WLS) were more likely to remain childless (see Figures 2, 3 and model 3 of Tables SM2–SM5). Among women, those higher educated remained childless more often in contrast to lower childlessness for those who never worked (i.e., no reported first occupation) or were employed in the service sector. Education and occupation did not influence childlessness in men.

### Variance Explained by PGSs

The effect sizes for the PGSs were modest: an increase of 1 SD in the AFB PGS increased the odds of remaining childless with 1.127, 1.226, 1.087, and 1.127 in the female HRS, female WLS, male HRS, and male WLS, respectively. However, in the models in which the socio-demographic factors were included these effects reduced to 1.026, 1.147, 1.105, and 0.977. This is relatively small compared to some socio-demographic factors, such as education, where a 1 SD increase in years of education resulted in an increase in the odds of remaining childless of 1.24, 1.53 in the female HRS and WLS samples, respectively. For those who married after age 36, the odds of remaining childless are 4.6, 10.8, 8.8, and 44.8 times higher than those who wed before the age 21, in the four samples, respectively. Examining the adjusted McFadden *R*^2^, the goodness of fit in the models with only genetic factors is markedly lower and even negative (<0.001) than models with socio-demographic factors (between 0.19 and 0.46).

### PGS for AFB Especially Relevant Among Women Who Married at Higher Ages

**Figure 4:**
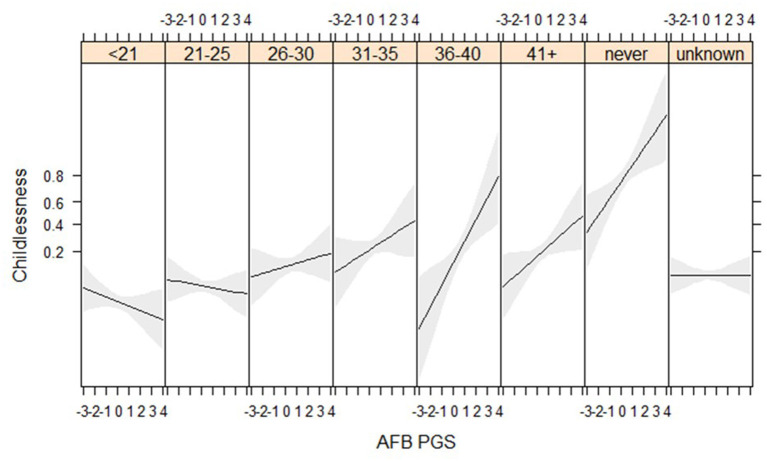
Age at first birth PGSs especially relevant among later married women in the HRS sample (results from the model in Table SM10).

We find suggestive evidence that PGSs for AFB are especially related to childlessness among women who married at higher ages (gene^*^socio-demographic interaction) (Figure 4, Tables SM10, SM11). Genes related to AFB do not seem to relate to childlessness among women who married before 30, but have a positive association for those who married after 30. We are only able to detect these relationships in the HRS data, where more respondents marry at later ages, although the interaction between AFB and age at marriage has a similar direction in the WLS sample (Figure SM6). We expected that the PGSs for AFB and NEB would show a stronger association with childlessness during the second demographic transition, but we did not find these interaction effects (Table SM10).

### Gene-Socio-Demographic Correlations in the Expected Direction and Mixed Results for the Genetic Correlations

The PGSs related to reproductive behavior (higher AFB, lower NEB) are related to higher education and a higher age at first marriage or never marrying (Table 1), which is in line with our expectations. We find this in both the HRS and the WLS sample and among men and women. The LD score correlation between education and AFB and NEB is even higher. In females, we also see a positive correlation between the AFB PGS with PCOS PGS but an unexpected negative correlation between the AFB PGS with endometriosis PGS (Table 1). PGSs for higher age at menarche and higher age of menopause are related to higher AFB PGS (Table 1). The results from the correlation between the genetic scores are comparable to the results based on LD-score regression (Table 1); almost all of the correlations are in the same direction, although the LD-score regression estimates are higher in most cases. For men, the results are almost all insignificant, it only seems to be the case that genes positively related to testicular germ cell cancer correlate positively to genes related to a higher age at first birth. We cannot calculate LD-score correlations for men due to small sample size and low number of SNPs.

### Mediation of AFB and NEB PGSs Effects by Education and Marriage

To further assess the interplay between the PGSs for reproductive behavior (AFB, NEB), those for the biological traits, and the socio-demographic factors, we applied a KHB mediation analysis. Results indicate that the effect sizes of the AFB and NEB PGSs decrease 25–170% by including education and age at marriage in the model (Table 2). On the other hand, the effect sizes do not substantially decrease when including the PGSs for the biological traits.

**Table 2:** Results from the Karlson-Breen-Holm mediation analysis, percentage confounding presented.

		**HRS women**		**WLS women**			**HRS men**		**WLS men**	
	**Confounder**	**%**	***p***	**%**	***p***	**Confounder**	**%**	***p***	**%**	***p***
AFB PGS	Genetic PCs	12.93	0.335	−4.35	0.518	Genetic PCs	−23.19	0.413	3.82	0.637
	Education years	43.73	0.000	25.67	0.000	Education years	6.97	0.208	13.21	0.002
	Age marriage	44.91	0.000	60.65	0.000	Age marriage	42.28	0.004	50.39	0.001
	Educ & Marriage	74.98	0.000	61.49	0.000	Educ and Marriage	83.96	0.000	57.44	0.000
	Endometriosis PGS	0.41	0.749	−0.28	0.774	Azoospermia PGS	−0.88	0.597	0.1	0.926
	Menarche PGS	3.06	0.310	4.53	0.113	TDS PGS	−0.35	0.773	0.51	0.547
	Menopause PGS	0.84	0.553	−1.1	0.437	TGCC PGS	−3.28	0.189	1.12	0.396
	PCOS PGS	0.75	0.579	0	0.979	Cryptorchidism PGS	−1.07	0.439	−0.08	0.876
	All PGSs	4.82	0.187	3.09	0.350	Hypospadias PGS	0.00	0.997	0.02	0.908
	All together	82.03	0.000	62.73	0.000	Infertility PGS	−0.60	0.589	−0.29	0.809
						All PGSs	−7.31	0.051	−0.01	0.995
						All together	39.19	0.010	52.61	0.001
NEB PGS	Genetic PCs	−19.33	0.409	2.89	0.948	Genetic PCs	−110.75	0.064	10.15	0.589
	Education years	6.10	0.179	51	0.005	Education years	1.21	0.392	2.97	0.392
	Age marriage	22.89	0.031	141.61	0.005	Age marriage	14.83	0.320	44.24	0.007
	Educ & Marriage	25.03	0.023	172.02	0.001	Educ & Marriage	45.43	0.002	51.47	0.000
	Endometriosis PGS	−0.51	0.416	0.58	0.840	Azoospermia PGS	0.18	0.733	0.01	0.995
	Menarche PGS	−0.37	0.580	4.71	0.294	TDS PGS	−0.23	0.785	−0.4	0.640
	Menopause PGS	−0.53	0.506	−0.66	0.691	TGCC PGS	−1.97	0.263	−0.31	0.739
	PCOS PGS	0.54	0.572	0.08	0.974	Cryptorchidism PGS	0.23	0.741	−0.04	0.903
	All PGSs	−0.27	0.868	4.9	0.418	Hypospadias PGS	0.00	0.997	−0.11	0.874
	All together	24.97	0.027	159.56	0.001	Infertility PGS	0.17	0.864	−2.31	0.242
						All PGSs	−7.31	0.051	−0.01	0.995
						All together	16.24	0.294	46.15	0.011

*We control for the first 20 principal components (PCs). p, p-value; PGS, polygenic risk scores; NEB, number ever born; AFB, age at first birth; Educ, Education years; PCOS, polycystic ovary syndrome; TDS, testicular dysgenesis syndrome; TGCC, testicular germ cell tumors. The effect of the NEB genetic score is mediated for more than 100% by the age at marriage and education. This is because the effect of this score reversed in direction after including marriage. The reversed effect of the NEB PGS is not significant, so this could be interpreted as complete mediation*.

### Sex Differences in Genetic and Socio-Demographic Influences

We found some support that particular PGSs differently associate to childlessness in men and women, although variation was small. The PGSs associated with later ages at menarche negatively relate to childlessness in men but positively relate to childlessness in women (WLS, see Table SM7). The PGS for a later age at menopause does not relate to childlessness in men but positively relates to childlessness probabilities in women. In the HRS we find no differences in the relationship of the PGSs and therefore the findings from the WLS should be interpreted with caution. For socio-demographic factors, education only influenced women but not men, in line with findings from previous studies. Never being married has an even stronger effect on childlessness in men than in women.

### Weaker Effects in the Black HRS Sample

We performed the same analyses in the sample of black respondents from the HRS. None of the PGSs have a significant association with childlessness in this sample, although the directions of the associations seem to be similar to those in the white sample (see Tables SM8, SM9). These differences are not likely due to sample size differences, since when we run the same analyses in a random selection of the same sample size in the white sample, the effects are similar to those described above in the full sample (results available upon request). However, we did not find a significant interaction between race and the AFB and NEB PGSs and the pseudo *R*^2^ did not largely differ between the white and black samples. The effect of marriage and birth year are significantly weaker in the black male sample compared to the white male sample.

## Conclusion and Discussion

### Main Findings

In this paper we apply an innovative and explorative approach to studying childlessness, in which we include PGS from a large range of fertility related outcomes in combination with sociodemographic factors. We find that socio-demographic factors explain 19–46% in childlessness while the current PGS explain <1% of the variance, and only PGSs from large GWASs are related to childlessness. Our findings also indicate that genetic and socio-demographic factors are not independent, with PGSs for AFB and NEB related to education and age at marriage. Socio-demographic factors will always be more important for these behavioral traits. The predictive power of the PGSs will remain lower, but as sample sizes for GWAS increase, we know that the number of loci discovered and predictive power of these scores will increase (Mills and Rahal, 2019).

### Replications

An important strength of this study is the use of two independent samples (HRS, WLS) for replication. Several findings replicated, such as the effects of the socio-demographic factors (education, age at marriage), the association of the PGS for AFB and NEB with childlessness and how effects are partly mediated by education and age at marriage. Other findings, however, did not replicate for substantive reasons related to the sample properties. Genes related to AFB were only important for women who married (and thus presumably tried to conceive) at older ages in the HRS, a finding that did not replicate in the WLS. This is likely related to the fact that only 3% (*n* = 140) of women married over the age of 31 in the WLS, making it underpowered to detect any effects, whereas this group was 12% (*n* = 826) in the HRS. Given the postponement of unions and childbearing in more recent cohorts, further tests are required.

Even though we perform a large number of test, we use a standard significance threshold of 0.05 for significance testing. We contend that our hypothesis driven tests, in combination with the replication in the two samples in both men and women applies sufficient caution against false positives. The replication of estimating the association of the PGSs with childlessness using different *p*-value cutoffs (as displayed in Figures SM2–SM5) serves as an additional robustness check. Findings that are not robust are expected to differ in direction and strength by using different *p*-value cutoffs. Here we find that AFB and NEB PGSs show associations in similar directions across *p*-value cutoff criteria while this is not the case for all PGSs for biological traits.

### Ancestral and Sex Differences

We found smaller and non-significant effects for the PGSs on the black individuals in the HRS, which shows that it is not advisable to apply the PGSs used in this study to non-European ancestry groups. This is due to fact around 90% of the GWASs are derived from European-ancestry populations (Mills and Rahal, 2019), including those used here. Due to patterns of human dispersal out of Africa, population structure and stratification, PGSs derived from one ancestry group cannot be applied to another. The greater genetic variation among black individuals calls for a specific ancestry group GWAS (Tishkoff et al., 2009). PGSs applied outside of non-European ancestry samples are incorrect and not reliable (Martin et al., 2017). These ancestral differences are not to be confused with self-reported race or ethnicity, which are socially constructed and not biological categories.

Regarding sex differences, we confirmed that education has a weaker influence on childlessness in men, while being married is more important for men. There is some evidence that genes related to certain biological traits (age at menarche and menopause, male infertility) have opposite effects in men and in women (in WLS only), in line with findings of genetic sexual dimorphism of childlessness (Verweij et al., 2017).

### Interpretations

The suggestive finding that genetic scores related to a higher age at first birth are more important for remaining childless among women who got married at higher ages needs replication and opens questions for further research. This AFB genetic score could be indicative of biologically having more difficulties in conceiving, in which case the interpretation would be that those who genetically are more likely to experience fecundity problems, and who postpone childbearing, are most likely to remain childless. On the other hand, the genetic score related to a higher age at first birth could also be indicative of having lower fertility desires, as previous studies found that fertility desires are partly heritable (Kohler et al., 1999; Miller et al., 2010) and that people who desire fewer children attempt to have children at higher ages (Miller et al., 2010). In this case it could be that those who have lower fertility desires and get married at higher ages are most likely to remain childless.

Our study showed that both higher education and age at marriage are correlated with higher PGS for AFB and lower PGS for NEB, indicating that the PGSs capture genes related to personal characteristics linked to childlessness (i.e., higher education, later partnering). It might also be that pleiotropic genetic effects that influence both childlessness and other outcomes (education, age at marriage) are at play or that genes causally related to educational attainment are correlated with AFB and NEB PGSs due to the large phenotypic relation between AFB, NEB and education. These findings add to the general idea that SNPs found in a GWAS might be associated with the trait of interest indirectly through other outcomes. The same holds for example for SNPs found for education, as effects within families are much smaller, indicating that part of the effect of genes related to education are confounded by family effects (Lee et al., 2018). The fact that we found that genetic and socio-demographic factors do not independently influence childlessness, underscores the importance of simultaneously examining their influences and adopting a sociogenomic approach.

Looking at genetic correlations, in line with an earlier study (Barban et al., 2016), we found an overlap in genes related to reproductive behavior (AFB, NEB) and biological infertility traits (endometriosis, PCOS). The positive genetic correlation between age at menarche, menopause and AFB was somewhat unexpected, but could be interpreted as one set of genes that delay biological maturation and development, resulting in an overall biological shift to fertility in later life (Mostafavi et al., 2017). The finding that a higher genetic risk for endometriosis goes along with a lower genetic propensity for a later age at first birth is unexpected, and since we find this only in our WLS sample this needs replication.

### Explanations for Small Effects of the PGSs

It is important to note that genetic factors for reproductive behavior explained <1% of the variation in childlessness, whereas twin studies suggested heritability to be between 20 and 50% (Verweij et al., 2017). The discrepancy between heritability and association-based studies has been related to the phenomena of missing (Manolio et al., 2009) and hidden heritability (Witte et al., 2014) which might be due to, for example, heterogeneity across the discovery samples (Tropf et al., 2017), the excluding of rare genetic variants or overestimation in twin studies (Yang et al., 2015). It may furthermore be methodological, since we add additional uncertainty by examining a phenotype (childlessness) different from the one in the GWAS discovery (AFB, NEB, endometriosis etcetera). That we only include genotyped SNPs and not imputed SNPs might be another reason, although previous research showed that this should not lead to a reduction in explained variance (Ware et al., 2017).

Another reason for the low explained variance is the sample sizes of GWASs, because as sample sizes are increasing, explained variance by PGSs is also increasing (Nolte et al., 2017), and therefore using a similar approach as used in this study will in the future likely entail stronger results. This is illustrated by for example research on educational attainment, where in 2013 a GWAS was conducted among 101,069 individuals, in which three significant SNPs were found (Rietveld et al., 2013). In 2016, 74 genome wide SNPs were identified when the sample size was expanded to 293,723 individuals (Okbay et al., 2016). In 2018, with a sample size of over a million individuals (*N* = 1,131,881) 1,271 SNPs were discovered in relation to educational attainment (Lee et al., 2018). The increase in the number of significant SNPs that are associated with an outcome also increase the explained variance by genetic scores, with 2.5–4% explained variance from the genetic scores from the 2013 GWAS, 6–7% explained variance from the 2016 GWAS and 11–13% from the most recent GWAS for educational attainment. Also for fertility related traits larger GWASs are or will be available, such as for endometriosis (Sapkota et al., 2017) and in the near future for AFB and NEB.

Although there have been considerable gains in prediction, we also note that the predictive power of polygenic scores does not increase in a linear manner as sample sizes grow. It may be that after a certain plateau is reached, such as SNP-heritability representing the actual ceiling of the genetic predisposition of a trait that can be achieved by GWAS, further increases in sample sizes may not yield better prediction. Polygenic scores may also be influenced by confounding factors such as environmental interactions or parental confounding (Kong et al., 2018). Instead of increasing sample size, it could be more useful to explore the biological function of the SNPs and genetic architecture in more depth.

The GWA studies for biological fecundity traits used the current study were based on very small samples. This could explain why we have many insignificant results for the PGSs for biological fecundity traits. The GWASs on which the results are based are relatively small, and arguably underpowered to detect any significant SNP effects (Rietveld et al., 2015). That we found no observed association of the age at menarche scores is unlikely to be due to power issues, given that the menarche PGS is based on a large sample GWAS. This could be related to the fact that menarche does not have a straightforward (phenotypic) association with fertility (Guldbrandsen et al., 2014). Unfortunately, the HRS and WLS did not include information about the actual biological fecundity traits on which we created PGSs (such as information on age at menarche, sperm count or PCOS). This would have been a valuable contribution to this study and including these traits will be an interesting venue for future research. At the same time, this is one of the promising features of sufficiently powered PGSs in the foreseeable future: even in the absence of measured phenotypes, PGSs can be used as genetic proxies in prediction models.

Another reason for the small effects of the PGSs is that our study does not distinguish between voluntary and involuntary childlessness, resulting in a heterogeneous phenotype. Would we be able to distinguish between different types of childlessness, it would be likely that PGSs for biological fecundity traits have a stronger effect on involuntary childlessness than on voluntary childlessness.

### Explanation of Null-Finding for the Interactions

We did not find that PGSs for reproductive behavior have a stronger association with childlessness in more recent cohorts, going against theoretical expectations that individual freedom and thus genetic propensity for individual preferences would be more important in more recent cohorts (Kohler et al., 2002; Briley et al., 2015; Tropf et al., 2015). Since GWA studies are conducted in large samples from a range of birth cohorts and contexts (Conley, 2017; Tropf et al., 2017), genetic studies conducted in different countries and birth cohorts only isolate a small portion of the genetic variants related to the trait, which are the ones that are, regardless of the environment, robustly associated with the trait (Tropf et al., 2017). Thus, we might not be detecting interactions between genes and socio-demographics, because the genes identified in the GWASs which form the basis of the genetic scores are rather independent of the environment.

### Summary

To summarize, our results show that genetic and socio-demographic factors are related to childlessness, and that these influences are not independent. We show that the explained variance by PGS at this point is limited. However, the large GWA studies on a growing number of traits leads us to anticipate that a sociogenomic approach could inform future research and will provide useful insights.

## Data Availability Statement

The Database of Genotypes and Phenotypes (dbGaP) data that support the findings of this study are publicly available from the WLS (dbGaP phs001157.v1.p1) and the HRS (dbGaP phs000428.v1.p1).

## Author Contributions

RV, MM, GS, GG, and HS worked on conception and design of the study, did the analyses and interpretation of analyses. RV wrote the first draft and together with MM, GS, IN, NB, FT, GG, and HS worked on revisions of this draft. DC, KA, KZ, NR, MD, CS, MH, and AD helped in the acquisition of the data and revision of the draft.

### Conflict of Interest

The authors declare that the research was conducted in the absence of any commercial or financial relationships that could be construed as a potential conflict of interest. The reviewer DB declared a past co-authorship with several of the authors GS, NB, and HS to the handling editor.
