# Supplementary material for: Using Polygenic Scores in Social Science Research: Unraveling Childlessness
Source: Front Sociol. 2019 Nov 22;4:74. doi: 10.3389/fsoc.2019.00074 (PMC8022451; doi:10.3389/fsoc.2019.00074)
Supplement: Supplementary file 1 [file Data_Sheet_1.pdf]

***Supplementary Material***

**Table SM 1** Descriptive statistics of the two samples

| | HRS women | HRS men | | WLS women | WLS men |
|---|---|---|---|---|---|
| N | 6,165 | 4,576 | N | 4,625 | 4,218 |
| Childless (%) | 10.8 | 12.5 | prop childless | 6.6 | 6.3 |
| Birth year (quartiles) | | | Birth year(quartiles) | | |
| Q1 | 1932 | 1932 | Q1 | 1939 | 1938 |
| mean | 1940 | 1939 | mean | 1939 | 1939 |
| Q3 | 1949 | 1949 | Q3 | 1939 | 1939 |
| Education (%) | | | Education years (%) | | |
| <High school | 16.6 | 15.6 | | | |
| High school | 38.9 | 32.3 | High school | 58.3 | 48.6 |
| Some college | 24.4 | 22.8 | Some college | 16.9 | 16.7 |
| >College | 19.9 | 29.3 | BSc | 13.8 | 15.3 |
| | | | >BSc | 11.0 | 19.5 |
| Age first marriage (%) | | | Age first marriage (%) | | |
| <21 | 30.6 | 13.3 | <21 | 37.8 | 11.6 |
| 21-25 | 24.3 | 36.2 | 21-25 | 47.4 | 57.3 |
| 26-30 | 7.3 | 14.5 | 26-30 | 7.6 | 19.9 |
| 31-35 | 3.9 | 5.9 | 31-35 | 1.7 | 4.3 |
| 36-40 | 3.3 | 3.1 | 36-40 | 0.5 | 1.1 |
| 41+ | 4.4 | 7.3 | 41+ | 0.8 | 1.3 |
| never married | 3.1 | 4.2 | never married | 3.5 | 3.6 |
| unknown age | 23.9 | 15.5 | unknown age | 0.7 | 0.9 |
| First occupation (%) | | | First occupation (%) | | |
| clerks | 22.9 | 4.8 | clerks | 44.5 | 6.2 |
| sales | 9.2 | 8.9 | sales | 3.3 | 4.9 |
| services | 13.1 | 5.1 | service | 11.9 | 2.4 |
| manager | 9.5 | 16.9 | administrators, managers | 2.0 | 6.5 |
| farming | 0.9 | 2.8 | farming | 0.9 | 7.7 |
| professionals | 16.1 | 15.6 | professional, technical | 22.6 | 26.4 |
| mechanics, production | 20.8 | 27.7 | manufacturing, construction | 9.5 | 42.3 |
| operators | 7.5 | 16.2 | no job | 5.4 | 3.6 |
| army | 0.1 | 2.0 | | | |
| Religion (%) | | | Religion (%) | | |
| not religious | 6.5 | 10.9 | not religious | 7.7 | 11.5 |
| Other | 1.0 | 1.0 | Other | 15.3 | 13.7 |
| Protestant | 61.6 | 57.1 | Protestant | 37.5 | 36.2 |
| Roman Catholic | 28.8 | 28.7 | Roman Catholic | 39.6 | 38.5 |
| Jewish | 2.1 | 2.4 | | | |

Note: for birth year we report the mean and the first and third quartile. In the WLS there is little variation in birth year due to the sampling design.

**Table SM 2**

Logistic regression results on childlessness in the female HRS sample.

| | Model 1 | | | Model 2 | | | Model 3 | | | Model 4 | | |
|---|---|---|---|---|---|---|---|---|---|---|---|---|
| | OR | 95%CI | p | OR | 95%CI | p | OR | 95%CI | p | OR | 95%CI | p |
| Intercept | 0.024 | (0.004-0.098) | 0.000 | 0.023 | (0.004-0.096) | 0.000 | 0.043 | (0.028-0.065) | 0.000 | 0.006 | (0.001-0.036) | 0.000 |
| PGS (standardized) | | | | | | | | | | | | |
| NEB | 0.861 | (0.748-0.992) | 0.038 | | | | | | | 0.869 | (0.741-1.019) | 0.082 |
| AFB | 1.127 | (1.017-1.249) | 0.028 | | | | | | | 1.026 | (0.912-1.155) | 0.748 |
| PCOS | | | | 1.095 | (0.91-1.317) | 0.367 | | | | 1.134 | (0.918-1.4) | 0.285 |
| Endometriosis | | | | 1.499 | (0.799-2.814) | 0.223 | | | | 1.478 | (0.724-3.021) | 0.312 |
| Menarche | | | | 1.081 | (0.981-1.192) | 0.110 | | | | 1.072 | (0.96-1.196) | 0.200 |
| Menopause | | | | 1.032 | (0.94-1.132) | 0.520 | | | | 1.078 | (0.970-1.199) | 0.162 |
| Socio-demographic | | | | | | | | | | | | |
| Birth year (standardized) | | | | | | | 1.34 | (1.205-1.49) | 0.000 | 1.36 | (1.221-1.515) | 0.000 |
| Education years (standardized) | | | | | | | 1.244 | (1.082-1.432) | 0.002 | 1.203 | (1.043-1.389) | 0.011 |
| Age first marriage (reference is before 21) | | | | | | | | | | | | |
| 21-25 | | | | | | | 1.47 | (1.081-2.009) | 0.012 | 1.429 | (1.048-1.955) | 0.019 |
| 26-30 | | | | | | | 3.091 | (2.158-4.417) | 0.000 | 3.047 | (2.121-4.366) | 0.000 |
| 31-35 | | | | | | | 6.319 | (4.288-9.279) | 0.000 | 6.363 | (4.302-9.377) | 0.000 |
| 36-40 | | | | | | | 4.598 | (2.794-7.386) | 0.000 | 4.662 | (2.818-7.531) | 0.000 |
| 41+ | | | | | | | 5.663 | (3.845-8.293) | 0.000 | 5.69 | (3.847-8.368) | 0.000 |
| never married | | | | | | | 120.26 | (74.901-200.1) | 0.000 | 122.4 | (75.96-204.70) | 0.000 |
| unknown age | | | | | | | 3.432 | (2.518-4.707) | 0.000 | 3.479 | (2.548-4.779) | 0.000 |

**Table SM 2**

Continued

| | Model 1 | | | Model 2 | | | Model 3 | | | Model 4 | | |
|---|---|---|---|---|---|---|---|---|---|---|---|---|
| | OR | 95%CI | p | OR | 95%CI | p | OR | 95%CI | p | OR | 95%CI | p |
| First occupation (reference are clerks) | | | | | | | | | | | | |
| army | | | | | | | 1.44 | (0.073-9.574) | 0.755 | 1.231 | (0.061-8.561) | 0.866 |
| farming | | | | | | | 1.633 | (0.57-3.937) | 0.325 | 1.569 | (0.541-3.83) | 0.380 |
| manager | | | | | | | 1.391 | (1.016-1.895) | 0.051 | 1.415 | (1.03-1.933) | 0.042 |
| mechanics, production | | | | | | | 1.016 | (0.753-1.369) | 0.960 | 1.028 | (0.76-1.387) | 0.916 |
| operators | | | | | | | 0.541 | (0.316-0.884) | 0.018 | 0.554 | (0.322-0.91) | 0.024 |
| professionals | | | | | | | 1.337 | (1.005-1.778) | 0.066 | 1.377 | (1.034-1.835) | 0.044 |
| sales | | | | | | | 1.008 | (0.702-1.429) | 0.901 | 1.023 | (0.711-1.453) | 0.956 |
| services | | | | | | | 0.714 | (0.496-1.013) | 0.065 | 0.729 | (0.506-1.038) | 0.085 |
| Religion (reference is not religious) | | | | | | | | | | | | |
| Other | | | | | | | 1.215 | (0.501-2.705) | 0.741 | 1.231 | (0.509-2.741) | 0.995 |
| Protestant | | | | | | | 0.923 | (0.668-1.297) | 0.976 | 0.982 | (0.704-1.39) | 0.982 |
| Roman Catholic | | | | | | | 0.977 | (0.686-1.407) | 0.774 | 0.96 | (0.667-1.397) | 0.391 |
| Jewish | | | | | | | 0.879 | (0.448-1.639) | 0.593 | 0.589 | (0.184-1.869) | 0.571 |
| McFadden R2 adj | 0.001/-0.001+ | | | 0.000/-0.003+ | | | 0.182 | | | 0.178 | | |
| N | 6165 | | | 6165 | | | 6133 | | | 6133 | | |

Notes: In model 1,2 and 4 we control for the first 20 genetic principal components (PCs). + R2 for model without and with PCs. The models with PCs have negative r2 because of the large number of coefficients and the poor fit. Abbreviations: OR, odds ratio; CI: confidence interval; p, p-value; PGS, polygenic risk score; NEB, number of children ever born, AFB; age at first birth; PCOS, polycystic ovary syndrome.

**Table SM 3**

Multilevel logistic regression results on childlessness in the female WLS sample.

| | Model 1 | | | Model 2 | | | Model 3 | | | Model 4 | | |
|---|---|---|---|---|---|---|---|---|---|---|---|---|
| | OR | 95%CI | p | OR | 95%CI | p | OR | 95%CI | p | OR | 95%CI | p |
| Intercept | 0.066 | (0.059-0.075) | 0.000 | 0.067 | (0.06-0.076) | 0.000 | 0.053 | (0.028-0.099) | 0.000 | 0.051 | (0.027-0.098) | 0.000 |
| PGS (standardized) | | | | | | | | | | | | |
| NEB | 1.002 | (0.876-1.147) | 0.976 | | | | | | | 1.057 | (0.877-1.274) | 0.559 |
| AFB | 1.226 | (1.074-1.339) | 0.000 | | | | | | | 1.147 | (0.962-1.367) | 0.127 |
| PCOS | | | | 1.007 | (0.832-1.220) | 0.893 | | | | 0.949 | (0.736-1.222) | 0.684 |
| Endometriosis | | | | 1.045 | (0.758-1.440) | 0.655 | | | | 1.035 | (0.68-1.577) | 0.871 |
| Menarche | | | | 1.086 | (0.953-1.237) | 0.114 | | | | 1.087 | (0.911-1.297) | 0.355 |
| Menopause | | | | 0.945 | (0.813-1.099) | 0.316 | | | | 0.768 | (0.619-0.954) | 0.018 |
| Socio-demographic | | | | | | | | | | | | |
| Birth year (standardized) | | | | | | | 0.972 | (0.82-1.153) | 0.749 | 0.986 | (0.829-1.173) | 0.874 |
| Education years (standardized) | | | | | | | 1.525 | (1.212-1.92) | 0.000 | 1.527 | (1.204-1.936) | 0.000 |
| Age at first marriage (reference is before 21) | | | | | | | | | | | | |
| 21-25 | | | | | | | 2.202 | (1.36-3.564) | 0.001 | 2.199 | (1.353-3.575) | 0.001 |
| 26-30 | | | | | | | 5.61 | (3.109-10.121) | 0.000 | 5.394 | (2.956-9.842) | 0.000 |
| 31-35 | | | | | | | 13.047 | (6.182-27.535) | 0.000 | 12.63 | (5.844-27.314) | 0.000 |
| 36-40 | | | | | | | 10.833 | (2.836-41.384) | 0.000 | 10.85 | (2.712-43.475) | 0.001 |
| 41+ | | | | | | | 59.403 | (25.389-138.9) | 0.000 | 73.84 | (30.13-180.95) | 0.000 |
| never married | | | | | | | 471.38 | (234.80-946.3) | 0.000 | 560.8 | (272.8-1152.8) | 0.000 |
| unknown age | | | | | | | 9.351 | (0.981-89.151) | 0.052 | 12.05 | (1.22-119.117) | 0.033 |

**Table SM 3**

Continued

| | Model 1 | | | Model 2 | | | Model 3 | | | Model 4 | | |
|---|---|---|---|---|---|---|---|---|---|---|---|---|
| | OR | 95%CI | p | OR | 95%CI | p | OR | 95%CI | p | OR | 95%CI | p |
| First occupation (reference are clerks) | | | | | | | | | | | | |
| administrators, managers | | | | | | | 1.309 | (0.526-3.256) | 0.563 | 1.351 | (0.535-3.412) | 0.524 |
| farming | | | | | | | 1.067 | (0.142-8.032) | 0.949 | 0.986 | (0.128-7.575) | 0.989 |
| manufacturing, construction | | | | | | | 1.124 | (0.61-2.071) | 0.709 | 1.172 | (0.633-2.168) | 0.614 |
| no job | | | | | | | 0.394 | (0.167-0.932) | 0.034 | 0.377 | (0.156-0.912) | 0.030 |
| professional, technical | | | | | | | 0.554 | (0.336-0.915) | 0.021 | 0.554 | (0.331-0.928) | 0.025 |
| sales | | | | | | | 0.554 | (0.176-1.749) | 0.314 | 0.502 | (0.154-1.638) | 0.254 |
| service | | | | | | | 0.58 | (0.296-1.137) | 0.113 | 0.505 | (0.248-1.031) | 0.061 |
| Religion (reference is not religious) | | | | | | | | | | | | |
| Other | | | | | | | 0.443 | (0.25-0.786) | 0.005 | 0.414 | (0.229-0.748) | 0.004 |
| Protestant | | | | | | | 0.376 | (0.217-0.652) | 0.001 | 0.334 | (0.188-0.595) | 0.000 |
| Roman Catholic | | | | | | | 0.286 | (0.163-0.502) | 0.000 | 0.277 | (0.155-0.495) | 0.000 |
| N individuals | 4625 | | | 4625 | | | 4327 | | | 4327 | | |
| N households | 3999 | | | 3999 | | | 3775 | | | 3775 | | |
| Household level variance | 0.000 | | | 0.000 | | | 0.000 | | | 0.000 | | |
| VPC (σ2μ0/( σ2μ0+3.29) | 0.000 | | | 0.000 | | | 0.000 | | | 0.000 | | |
| McFadden R2 adj | 0.005/0.000+ | | | -0.001/-0.007+ | | | 0.424 | | | 0.418 | | |

Notes: In model 1,2 and 4 we control for the first 20 genetic principal components (PCs). + R2 for model without and with PCs. The models with PCs have negative r2 because of the large number of coefficients and the poor fit. Abbreviations: OR, odds ratio; CI: confidence interval; p, p-value; PGS, polygenic risk score; NEB, number of children ever born, AFB; age at first birth; PCOS, polycystic ovary syndrome; VPC, variance partitioning coefficient.

**Table SM 4**

Logistic regression results on childlessness in the male HRS sample.

| | Model 1 | | | Model 2 | | | Model 3 | | | Model 4 | | |
|---|---|---|---|---|---|---|---|---|---|---|---|---|
| | OR | 95%CI | p | OR | 95%CI | p | OR | 95%CI | p | OR | 95%CI | p |
| Intercept | 0.103 | (0.038-0.173) | 0.000 | 0.099 | (0.036-0.168) | 0.000 | 0.029 | (0.014-0.056) | 0.000 | 0.012 | (0.003-0.034) | 0.000 |
| PGS (standardized) | | | | | | | | | | | | |
| NEB | 0.873 | (0.749-1.018) | 0.067 | | | | | | | 0.847 | (0.706-1.018) | 0.071 |
| AFB | 1.078 | (0.962-1.209) | 0.232 | | | | | | | 1.105 | (0.962-1.27) | 0.198 |
| TDS | | | | 1.157 | (0.901-1.488) | 0.218 | | | | 1.021 | (0.761-1.37) | 0.831 |
| Azoospermia | | | | 1.030 | (0.941-1.127) | 0.525 | | | | 1.014 | (0.911-1.128) | 0.800 |
| TGCC | | | | 0.787 | (0.61-1.015) | 0.063 | | | | 0.825 | (0.61-1.115) | 0.230 |
| Infertility (low sperm count) | | | | 1.086 | (0.798-1.478) | 0.669 | | | | 1.213 | (0.844-1.741) | 0.319 |
| Cryptorchidism | | | | 0.848 | (0.67-1.074) | 0.157 | | | | 0.783 | (0.592-1.035) | 0.071 |
| Hypospadias | | | | 0.974 | (0.86-1.104) | 0.559 | | | | 1.008 | (0.869-1.171) | 0.908 |
| Socio-demographic | | | | | | | | | | | | |
| Birth year (standardized) | | | | | | | 1.709 | (1.519-1.926) | 0.000 | 1.744 | (1.546-1.97) | 0.000 |
| Education years (standardized) | | | | | | | 1.06 | (0.909-1.24) | 0.290 | 1.018 | (0.87-1.194) | 0.570 |
| Age first marriage (reference is before 21) | | | | | | | | | | | | |
| 21-25 | | | | | | | 2.018 | (1.24-3.454) | 0.009 | 2.000 | (1.227-3.431) | 0.010 |
| 26-30 | | | | | | | 3.93 | (2.361-6.847) | 0.000 | 3.814 | (2.283-6.665) | 0.000 |
| 31-35 | | | | | | | 6.839 | (3.949-12.288) | 0.000 | 6.856 | (3.936-12.381) | 0.000 |
| 36-40 | | | | | | | 8.784 | (4.713-16.701) | 0.000 | 9.324 | (4.961-17.867) | 0.000 |
| 41+ | | | | | | | 8.741 | (5.103-15.578) | 0.000 | 8.76 | (5.09-15.68) | 0.000 |
| never married | | | | | | | 229.77 | (123.04-454.0) | 0.000 | 256.5 | (135.99-511.8) | 0.000 |
| unknown age | | | | | | | 8.906 | (5.337-15.576) | 0.000 | 9.159 | (5.462-16.086) | 0.000 |

**Table SM 4**

Continued

| | Model 1 | | | Model 2 | | | Model 3 | | | Model 4 | | |
|---|---|---|---|---|---|---|---|---|---|---|---|---|
| | OR | 95%CI | p | OR | 95%CI | p | OR | 95%CI | p | OR | 95%CI | p |
| First occupation (reference are clerks) | | | | | | | | | | | | |
| army | | | | | | | 1.114 | (0.461-2.534) | 0.908 | 1.186 | (0.488-2.717) | 0.995 |
| farming | | | | | | | 0.699 | (0.233-1.824) | 0.477 | 0.755 | (0.25-1.988) | 0.591 |
| manager | | | | | | | 0.924 | (0.543-1.625) | 0.686 | 0.92 | (0.538-1.621) | 0.670 |
| mechanics, production | | | | | | | 1.152 | (0.699-1.972) | 0.645 | 1.197 | (0.723-2.056) | 0.542 |
| operators | | | | | | | 1.294 | (0.763-2.27) | 0.351 | 1.341 | (0.786-2.365) | 0.289 |
| professionals | | | | | | | 1.245 | (0.736-2.179) | 0.505 | 1.303 | (0.765-2.291) | 0.402 |
| sales | | | | | | | 0.751 | (0.409-1.398) | 0.288 | 0.758 | (0.411-1.418) | 0.311 |
| services | | | | | | | 1.063 | (0.545-2.08) | 0.883 | 1.149 | (0.584-2.263) | 0.703 |
| Religion (reference is not religious) | | | | | | | | | | | | |
| Other | | | | | | | 1.912 | (0.829-4.152) | 0.245 | 2.017 | (0.858-4.481) | 0.320 |
| Protestant | | | | | | | 0.842 | (0.624-1.149) | 0.305 | 0.865 | (0.633-1.195) | 0.292 |
| Roman Catholic | | | | | | | 0.854 | (0.605-1.211) | 0.071 | 0.833 | (0.576-1.209) | 0.157 |
| Jewish | | | | | | | 0.466 | (0.192-1.012) | 0.106 | 0.416 | (0.125-1.354) | 0.082 |
| McFadden R2 adj | -0.001/-0.003+ | | | -0.003/-0.005+ | | | 0.236 | | | 0.232 | | |
| N | 4576 | | | 4576 | | | 4551 | | | 4551 | | |

Notes: In model 1,2 and 4 we control for the first 20 genetic principal components (PCs). + R2 for model without and with PCs. The models with PCs have negative r2 because of the large number of coefficients and the poor fit. Abbreviations: OR, odds ratio; CI: confidence interval; p, p-value; PGS, polygenic risk score; NEB, number of children ever born, AFB; age at first birth; TDS, testicular dysgenesis syndrome; TGCC, testicular germ cell tumors

**Table SM 5**

Multilevel logistic regression results on childlessness in the male WLS sample.

| | Model 1 | | | Model 2 | | | Model 3 | | | Model 4 | | |
|---|---|---|---|---|---|---|---|---|---|---|---|---|
| | OR | 95%CI | p | OR | 95%CI | p | OR | 95%CI | p | OR | 95%CI | p |
| Intercept | 0.064 | (0.056-0.074) | 0.000 | 0.063 | (0.055-0.073) | 0.000 | 0.009 | (0.002-0.045) | 0.000 | 0.007 | (0.001-0.035) | 0.000 |
| PGS (standardized) | | | | | | | | | | | | |
| NEB | 0.911 | (0.787-1.055) | 0.213 | | | | | | | 0.899 | (0.726-1.113) | 0.329 |
| AFB | 1.127 | (0.987-1.286) | 0.078 | | | | | | | 0.977 | (0.806-1.183) | 0.809 |
| TDS | | | | 0.855 | (0.618-1.182) | 0.342 | | | | 0.885 | (0.559-1.403) | 0.605 |
| Azoospermia | | | | 0.918 | (0.809-1.043) | 0.188 | | | | 0.808 | (0.669-0.975) | 0.026 |
| TGCC | | | | 1.156 | (0.922-1.449) | 0.209 | | | | 1.092 | (0.791-1.506) | 0.593 |
| Infertility (low sperm count) | | | | 1.266 | (1.036-1.548) | 0.021 | | | | 0.983 | (0.739-1.308) | 0.908 |
| Cryptorchidism | | | | 0.99 | (0.811-1.207) | 0.919 | | | | 1.054 | (0.798-1.392) | 0.712 |
| Hypospadias | | | | 1.000 | (0.872-1.147) | 0.999 | | | | 1.06 | (0.869-1.292) | 0.567 |
| Socio-demographic | | | | | | | | | | | | |
| Birth year (standardized) | | | | | | | 1.058 | (0.889-1.258) | 0.528 | 1.054 | (0.883-1.257) | 0.561 |
| Education years (standardized) | | | | | | | 0.996 | (0.783-1.265) | 0.971 | 1.026 | (0.803-1.311) | 0.836 |
| Age first marriage (reference is before 21) | | | | | | | | | | | | |
| 21-25 | | | | | | | 4.179 | (1.004-17.384) | 0.049 | 4.204 | (1.006-17.57) | 0.049 |
| 26-30 | | | | | | | 10.98 | (2.623-45.952) | 0.001 | 10.96 | (2.60-46.18) | 0.001 |
| 31-35 | | | | | | | 27.195 | (6.228-118.74) | 0.000 | 26.64 | (6.04-117.46) | 0.000 |
| 36-40 | | | | | | | 44.831 | (8.782-228.86) | 0.000 | 55.62 | (10.69-289.11) | 0.000 |
| 41+ | | | | | | | 90.207 | (19.24-422.91) | 0.000 | 92.13 | (19.28-440.23) | 0.000 |
| never married | | | | | | | 1896.8 | (421.03-8545) | 0.000 | 2529 | (547.8-1167.) | 0.000 |
| unknown age | | | | | | | 58.994 | (8.62-403.716) | 0.000 | 59.95 | (8.428-426.53) | 0.000 |

**Table SM 5**

Continued

| | Model 1 | | | Model 2 | | | Model 3 | | | Model 4 | | |
|---|---|---|---|---|---|---|---|---|---|---|---|---|
| | OR | 95%CI | p | OR | 95%CI | p | OR | 95%CI | p | OR | 95%CI | p |
| First occupation (reference are clerks) | | | | | | | | | | | | |
| administrators, managers | | | | | | | 0.981 | (0.381-2.526) | 0.968 | 1.091 | (0.415-2.87) | 0.860 |
| farming | | | | | | | 0.856 | (0.337-2.175) | 0.744 | 0.989 | (0.384-2.546) | 0.981 |
| manufacturing, construction | | | | | | | 0.745 | (0.355-1.563) | 0.436 | 0.837 | (0.393-1.784) | 0.646 |
| no job | | | | | | | 0.682 | (0.205-2.273) | 0.533 | 0.726 | (0.216-2.442) | 0.605 |
| professional, technical | | | | | | | 1.024 | (0.461-2.272) | 0.954 | 1.121 | (0.5-2.516) | 0.781 |
| sales | | | | | | | 0.52 | (0.159-1.696) | 0.278 | 0.518 | (0.151-1.774) | 0.295 |
| service | | | | | | | 0.331 | (0.059-1.856) | 0.209 | 0.381 | (0.067-2.176) | 0.278 |
| Religion (reference is not religious) | | | | | | | | | | | | |
| Other | | | | | | | 0.635 | (0.358-1.126) | 0.120 | 0.678 | (0.376-1.223) | 0.197 |
| Protestant | | | | | | | 0.535 | (0.311-0.922) | 0.024 | 0.537 | (0.304-0.947) | 0.032 |
| Roman Catholic | | | | | | | 0.504 | (0.296-0.857) | 0.011 | 0.496 | (0.286-0.861) | 0.013 |
| N individuals | 4218 | | | 4218 | | | 3957 | | | 3957 | | |
| N households | 3742 | | | 3742 | | | 3537 | | | 3537 | | |
| Household level variance | 0.231 | | | 0.221 | | | 0.114 | | | 0.107 | | |
| VPC (σ2μ0/( σ2μ0+3.29) | 0.066 | | | 0.063 | | | 0.034 | | | 0.032 | | |
| McFadden R2 adj | 0.002/-0.011+ | | | -0.002/-0.013+ | | | 0.449 | | | 0.438 | | |

Notes: In model 1,2 and 4 we control for the first 20 genetic principal components (PCs). + R2 for model with and without PCs. The models with PCs have negative r2 because of the large number of coefficients and the poor fit. Abbreviations: OR, odds ratio; CI: confidence interval; p, p-value; PGS, polygenic risk score; NEB, number of children ever born, AFB; age at first birth; TDS, testicular dysgenesis syndrome; TGCC, testicular germ cell tumors; VPC, variance partitioning coefficient.

**Table SM 6**

Sample size and effect of the PGSs

| Women | | | | | | | | |
|---|---|---|---|---|---|---|---|---|
| PGS | N GWAS | nr SNPs GWAS | SNPs HRS | SNPs WLS | bHRS | pHRS | bWLS | pWLS |
| AFB | 251,151 | 2,470,767 | 223,702 | 204,518 | 0.144 | **0.004** | 0.203 | **0.001** |
| NEB | 343,072 | 2,474,038 | 223,874 | 205,336 | -0.187 | **0.008** | -0.057 | 0.421 |
| PCOS | 3,930 | 965,435 | 91,618 | 88,052 | 0.093 | 0.322 | 0.005 | 0.943 |
| Endometriosis | 10,254 | 7,531,796 | 219,436 | 196,652 | 0.415 | 0.320 | 0.036 | 0.839 |
| EndometriosisB+ | 8,424 | 7,528,702 | 218,924 | 196,378 | 0.099 | 0.636 | -0.097 | 0.448 |
| Menarche | 329,345 | 10,562,209 | 218,050 | 195,429 | 0.078 | 0.114 | 0.081 | 0.220 |
| Menopause | 69,360 | 2,383,153 | 179,138 | 191,400 | 0.033 | 0.483 | -0.053 | 0.491 |
| Men | | | | | | | | |
| PGS | N GWAS | nr SNPs GWAS | SNPs HRS | SNPs WLS | bHRS | pHRS | bWLS | pWLS |
| AFB | 251,151 | 2,470767 | 223,702 | 204,518 | 0.099 | 0.077 | 0.164 | **0.018** |
| NEB | 343,072 | 2,474038 | 223,874 | 205,336 | -0.16 | **0.035** | -0.150 | **0.050** |
| TDS | 927 | 626,955 | 134,084 | 125,454 | -0.010 | 0.899 | 0.057 | 0.415 |
| Azoospermia | 132 | 324,453 | 133,216 | 121,922 | 0.028 | 0.529 | -0.106 | 0.122 |
| Azoospermia++ | 172 | 324,471 | 133,100 | 121,761 | 0.037 | 0.503 | -0.085 | 0.219 |
| TGCC | 651 | 626,955 | 134,366 | 125,754 | -0.105 | 0.158 | 0.077 | 0.290 |
| Infertility | 546 | 626,955 | 134,072 | 125,242 | 0.145 | 0.189 | 0.138 | 0.084 |
| Cryptorchidism | 577 | 629,955 | 134,110 | 125,522 | -0.073 | 0.369 | 0.010 | 0.897 |
| Hypospadias | 470 | 626,955 | 130,344 | 121,046 | -0.009 | 0.878 | 0.010 | 0.883 |

Notes: N GWAS are the number of participants in the GWAS study, nr SNPs GWAS are the number of SNPs reported in the result file from the original GWAS. SNPs HRS are the number of SNPs included in the PGS with p-value ≤1 in HRS, SNPs WLS are the number of SNPs included in the PGS with p-value ≤1 in WLS. bHRS and pHRS are the b-value and p-value of the effect in the HRS sample, including only 1 PGS and 20 principal components, bWLS and pWLS are the b-value and p-value of the effect in the WLS sample, including only 1 PGS and 20 principal components. +EndometriosisB reflects the more severe form of the disease. ++The normal azoospermia GWAS used compares individuals with azoospermia with those with normal sperm count, the second azoospermia compares them to men with normal sperm count and those with oligozoospermia. Abbreviations: PGS, polygenic risk scores; NEB, number of ever born children, AFB, age at first birth; PCOS, polycystic ovary syndrome; TDS, testicular dysgenesis syndrome; TGCC, testicular germ cell tumors. Significant p-values ($p<0.05$) are shown in bold.

.

**Table SM 7**

Multilevel models on childlessness on individuals clustered in households, with interactions by gender

| | HRS | | | WLS | | |
|---|---|---|---|---|---|---|
| | OR | 95%CI | p | OR | 95%CI | p |
| Intercept | 0.009 | (0.002-0.035) | 0.000 | 0.008 | (0.002-0.039) | 0.000 |
| PGS (standardized) | | | | | | |
| NEB | 0.889 | (0.724-1.093) | 0.268 | 0.91 | (0.74-1.119) | 0.383 |
| AFB | 1.116 | (0.953-1.307) | 0.202 | 0.975 | (0.806-1.179) | 0.803 |
| PCOS | 1.026 | (0.805-1.309) | 0.858 | 0.804 | (0.636-1.017) | 0.071 |
| Endometriosis | 1.74 | (0.842-3.598) | 0.107 | 0.997 | (0.673-1.478) | 0.949 |
| Menarche | 1.009 | (0.871-1.168) | 0.911 | 0.855 | (0.71-1.031) | 0.099 |
| Menopause | 1.02 | (0.873-1.191) | 0.861 | 1.057 | (0.862-1.295) | 0.595 |
| TDS | 1.025 | (0.737-1.425) | 0.851 | 0.864 | (0.588-1.269) | 0.555 |
| Azoospermia | 1.006 | (0.89-1.136) | 0.937 | 0.821 | (0.681-0.989) | 0.036 |
| TGCC | 0.837 | (0.596-1.174) | 0.343 | 1.087 | (0.805-1.468) | 0.602 |
| Infertility | 1.248 | (0.831-1.875) | 0.286 | 1.003 | (0.769-1.31) | 1.000 |
| Cryptorchidism | 0.755 | (0.551-1.036) | 0.067 | 1.075 | (0.889-1.301) | 0.554 |
| Hypospadias | 1.006 | (0.847-1.195) | 0.920 | 1.141 | (1.001-1.3) | 0.518 |
| Socio-demographic | | | | | | |
| Birth year (standardized) | 1.786 | (1.555-2.051) | 0.000 | 1.062 | (0.891-1.265) | 0.485 |
| Gender (reference is male) | 1.43 | (0.495-4.134) | 0.613 | 6.207 | (1.118-34.457) | 0.037 |
| Education years (standardized) | 1.016 | (0.849-1.217) | 0.624 | 1.023 | (0.803-1.304) | 0.859 |
| Age first marriage (reference is before 21) | | | | | | |
| 21-25 | 1.949 | (1.125-3.377) | 0.020 | 4.026 | (0.966-16.779) | 0.055 |
| 26-30 | 3.836 | (2.147-6.851) | 0.000 | 10.989 | (2.619-46.104) | 0.001 |
| 31-35 | 6.757 | (3.583-12.745) | 0.000 | 27.634 | (6.307-121.074) | 0.000 |
| 36-40 | 9.221 | (4.486-18.954) | 0.000 | 50.18 | (9.72-259.067) | 0.000 |
| 41+ | 9.056 | (4.894-16.759) | 0.000 | 88.417 | (18.672-418.674) | 0.000 |
| never married | 305.777 | (148.668-628.917) | 0.000 | 2270.972 | (498.53-10345.0) | 0.000 |
| unknown age | 9.659 | (5.357-17.415) | 0.000 | 55.422 | (8.046-381.738) | 0.000 |

**Table SM 7**
Continued

| | HRS | | | WLS | | |
|---|---|---|---|---|---|---|
| | OR | 95%CI | p | OR | 95%CI | p |
| First occupation (reference are clerks) | | | | | | |
| administrators, managers | | | | 1067.000 | (0.414-2.755) | 0.886 |
| farming | 0.776 | (0.25-2.405) | 0.659 | 0.978 | (0.384-2.495) | 0.981 |
| manufacturing, construction | | | | 0.828 | (0.395-1.737) | 0.622 |
| no job | | | | 0.731 | (0.217-2.463) | 0.615 |
| professional, technical | 1.3 | (0.695-2.431) | 0.474 | 1.087 | (0.492-2.401) | 0.824 |
| sales | 0.778 | (0.387-1.565) | 0.410 | 0.539 | (0.16-1.813) | 0.316 |
| service | 1.106 | (0.513-2.388) | 0.814 | 0.347 | (0.059-2.048) | 0.243 |
| army | 1.271 | (0.479-3.375) | 0.911 | | | |
| mechanics, production | 1.172 | (0.646-2.125) | 0.637 | | | |
| operators | 1.366 | (0.73-2.559) | 0.321 | | | |
| managers | 0.912 | (0.486-1.713) | 0.706 | | | |
| Religion (reference is not religious) | | | | | | |
| Other | 2.272 | (0.845-6.106) | 0.097 | 0.65 | (0.363-1.164) | 0.138 |
| Protestant | 0.871 | (0.605-1.253) | 0.413 | 0.515 | (0.296-0.899) | 0.019 |
| Roman Catholic | 0.822 | (0.54-1.251) | 0.317 | 0.511 | (0.297-0.877) | 0.015 |
| Jewish | 0.427 | (0.124-1.477) | 0.188 | | | |
| Interaction with gender (reference is male) | | | | | | |
| PGS * gender | | | | | | |
| NEB | 0.961 | (0.734-1.258) | 0.763 | 1.141 | (0.869-1.499) | 0.342 |
| AFB | 0.929 | (0.756-1.142) | 0.492 | 1.2 | (0.928-1.552) | 0.164 |
| PCOS | 1.133 | (0.858-1.496) | 0.380 | 1.212 | (0.926-1.587) | 0.162 |
| Endometriosis | 0.895 | (0.415-1.931) | 0.729 | 1.125 | (0.72-1.758) | 0.604 |
| Menarche | 1.065 | (0.879-1.291) | 0.506 | 1.253 | (0.972-1.616) | 0.082 |
| Menopause | 1.057 | (0.875-1.276) | 0.511 | 0.78 | (0.597-1.02) | 0.070 |
| TDS | 0.899 | (0.582-1.391) | 0.589 | 1.31 | (0.731-2.347) | 0.364 |
| Azoospermia | 0.998 | (0.851-1.17) | 0.997 | 1.165 | (0.909-1.492) | 0.228 |
| TGCC | 1.223 | (0.776-1.925) | 0.410 | 0.919 | (0.601-1.405) | 0.697 |
| Infertility | 0.761 | (0.439-1.319) | 0.354 | 0.752 | (0.522-1.085) | 0.128 |
| Cryptorchidism | 1.347 | (0.883-2.054) | 0.134 | 0.985 | (0.685-1.416) | 0.933 |
| Hypospadias | 1.048 | (0.833-1.32) | 0.607 | 1.13 | (0.871-1.465) | 0.356 |
| Socio-demographic | | | | | | |
| Birth year * gender | 0.768 | (0.641-0.919) | 0.005 | 0.934 | (0.73-1.195) | 0.578 |
| Education years * gender | 1.164 | (0.917-1.477) | 0.307 | 1.504 | (1.073-2.11) | 0.018 |

**Table SM 7**
Continued

| | HRS | | | WLS | | |
|---|---|---|---|---|---|---|
| | OR | 95%CI | p | OR | 95%CI | p |
| Age first marriage (reference is before 21) * gender | | | | | | |
| 21-25 | 0.756 | (0.397-1.441) | 0.445 | 0.533 | (0.118-2.405) | 0.410 |
| 26-30 | 0.835 | (0.412-1.69) | 0.635 | 0.492 | (0.104-2.329) | 0.371 |
| 31-35 | 0.963 | (0.443-2.091) | 0.951 | 0.458 | (0.086-2.428) | 0.368 |
| 36-40 | 0.505 | (0.203-1.258) | 0.177 | 0.236 | (0.028-2.002) | 0.195 |
| 41+ | 0.619 | (0.294-1.302) | 0.184 | 0.674 | (0.113-4.031) | 0.652 |
| never married | 0.519 | (0.209-1.29) | 0.182 | 0.235 | (0.044-1.256) | 0.095 |
| unknown age | 0.381 | (0.193-0.752) | 0.010 | 0.199 | (0.01-3.938) | 0.288 |
| First occupation (reference are clerks) * gender | | | | | | |
| administrators, managers | | | | 1.281 | (0.34-4.833) | 0.725 |
| farming | 1.855 | (0.385-8.923) | 0.448 | 1.149 | (0.123-10.745) | 0.899 |
| manufacturing, construction | | | | 1.446 | (0.555-3.764) | 0.445 |
| no job | | | | 0.548 | (0.123-2.437) | 0.428 |
| professional, technical | 1.065 | (0.525-2.161) | 0.837 | 0.522 | (0.203-1.342) | 0.174 |
| sales | 1.315 | (0.585-2.955) | 0.484 | 0.916 | (0.169-4.951) | 0.933 |
| service | 0.672 | (0.282-1.601) | 0.378 | 1.554 | (0.23-10.524) | 0.645 |
| army | 0.929 | (0.048-18.011) | 0.971 | | | |
| mechanics, production | 0.85 | (0.428-1.687) | 0.651 | | | |
| operators | 0.413 | (0.178-0.959) | 0.037 | | | |
| managers | 1577.000 | (0.763-3.257) | 0.209 | | | |
| Religion (reference is not religious) * gender | | | | | | |
| Other | 0.565 | (0.142-2.244) | 0.564 | 0.67 | (0.293-1.53) | 0.346 |
| Protestant | 1.132 | (0.671-1.911) | 0.515 | 0.71 | (0.323-1.561) | 0.392 |
| Roman Catholic | 1.154 | (0.647-2.058) | 0.668 | 0.531 | (0.242-1.165) | 0.112 |
| Jewish | 1.367 | (0.322-5.811) | 0.426 | | | |
| N | 10686 | | | 8284 | | |
| N households | 7568 | | | 6423 | | |
| Household level variance | 2.016 | | | 0.033 | | |
| VPC (σ2μ0/( σ2μ0+3.29) | 0.379 | | | 0.01 | | |
| McFadden R2 adj+ | 0.207 | | | 0.431 | | |

Notes: We control for the first 20 genetic principal components. Abbreviations: OR, odds ratio; CI: confidence interval; p, p-value; PGS, polygenic risk scores; NEB, number ever born, AFB; age at first birth; PCOS, polycystic ovary syndrome; TDS, testicular dysgenesis syndrome; TGCC, testicular germ cell tumors; VPC, variance partitioning coefficient.

**Table SM 8**

Logistic regression results on childlessness in the black female HRS sample

| | Model 1 | | | Model 2 | | | Model 3 | | | Model 4 | | | Diff white sample |
|---|---|---|---|---|---|---|---|---|---|---|---|---|---|
| | OR | 95%CI | p | OR | 95%CI | p | OR | 95%CI | p | OR | 95%CI | p | p |
| Intercept | 0.111 | (0.036-0.311) | 0.000 | 0.105 | (0.037-0.326) | 0.000 | 0.047 | (0.016-0.139) | 0.000 | 0.057 | (0.011-0.298) | 0.000 | |
| PGS (standardized) | | | | | | | | | | | | | |
| NEB | 0.802 | (0.538-1.194) | 0.352 | | | | | | | 0.863 | (0.565-1.319) | 0.604 | 0.973 |
| AFB | 1.206 | (0.907-1.602) | 0.172 | | | | | | | 1.16 | (0.855-1.575) | 0.313 | 0.268 |
| PCOS | | | | 1.214 | (0.832-1.771) | 0.274 | | | | 1.162 | (0.777-1.74) | 0.432 | 0.850 |
| Endometriosis | | | | 1.041 | (0.246-4.397) | 0.733 | | | | 1.167 | (0.258-5.277) | 0.626 | 0.587 |
| Menarche | | | | 0.85 | (0.633-1.141) | 0.585 | | | | 0.833 | (0.611-1.136) | 0.747 | 0.526 |
| Menopause | | | | 0.859 | (0.643-1.147) | 0.306 | | | | 0.840 | (0.650-1.200) | 0.430 | 0.497 |
| Socio-demographic | | | | | | | | | | | | | |
| Birth year (standardized) | | | | | | | 0.703 | (0.564-0.876) | 0.003 | 0.716 | (0.572-0.896) | 0.007 | 0.000 |
| Education years (standardized) | | | | | | | 1.491 | (1.169-1.902) | 0.003 | 1.44 | (1.122-1.849) | 0.011 | 0.211 |

**Table SM 8**

Continued

| | Model 1 | | | Model 2 | | | Model 3 | | | Model 4 | | | Diff white sample |
|---|---|---|---|---|---|---|---|---|---|---|---|---|---|
| | OR | 95%CI | p | OR | 95%CI | p | OR | 95%CI | p | OR | 95%CI | p | p |
| Age first marriage (reference is before 21) | | | | | | | | | | | | | |
| 21-25 | | | | | | | 1.715 | (0.761-3.871) | 0.183 | 1.673 | (0.734-3.815) | 0.213 | 0.587 |
| 26-30 | | | | | | | 4.228 | (1.796-9.953) | 0.001 | 3.876 | (1.606-9.358) | 0.001 | 0.364 |
| 31-35 | | | | | | | 3.645 | (1.443-9.209) | 0.012 | 3.751 | (1.455-9.669) | 0.000 | 0.407 |
| 36-40 | | | | | | | 7.573 | (3.001-19.11) | 0.000 | 8.633 | (3.352-22.234) | 0.000 | 0.192 |
| 41+ | | | | | | | 7.599 | (3.027-19.08) | 0.000 | 8.557 | (3.314-22.094) | 0.000 | 0.241 |
| never married | | | | | | | 8.546 | (4.067-17.957) | 0.000 | 9.395 | (4.405-20.038) | 0.000 | 0.000 |
| unknown age | | | | | | | 1.823 | (0.825-4.028) | 0.120 | 1.889 | (0.846-4.214) | 0.096 | 0.383 |
| First occupation (reference are clerks) | | | | | | | | | | | | | |
| army | | | | | | | 6.377 | (0.336-120.978) | 0.188 | 7.587 | (0.327-176.109) | 0.223 | 0.356 |
| farming | | | | | | | 1.68 | (0.176-16.035) | 0.649 | 1.321 | (0.129-13.539) | 0.807 | 0.877 |
| manager | | | | | | | 0.866 | (0.361-2.084) | 0.830 | 0.775 | (0.31-1.935) | 0.680 | 0.234 |
| mechanics, production | | | | | | | 0.757 | (0.392-1.467) | 0.465 | 0.803 | (0.408-1.58) | 0.488 | 0.502 |
| operators | | | | | | | 0.623 | (0.302-1.287) | 0.227 | 0.62 | (0.295-1.306) | 0.182 | 0.997 |
| professionals | | | | | | | 0.731 | (0.397-1.346) | 0.337 | 0.731 | (0.388-1.376) | 0.374 | 0.073 |
| sales | | | | | | | 0.925 | (0.374-2.285) | 0.953 | 0.983 | (0.389-2.485) | 0.971 | 0.708 |
| services | | | | | | | 0.557 | (0.311-1.003) | 0.052 | 0.542 | (0.297-0.988) | 0.040 | 0.444 |

**Table SM 8**

Continued

| | Model 1 | | | Model 2 | | | Model 3 | | | Model 4 | | | Diff white sample |
|---|---|---|---|---|---|---|---|---|---|---|---|---|---|
| | OR | 95%CI | p | OR | 95%CI | p | OR | 95%CI | p | OR | 95%CI | p | p |
| Religion (reference is not religious) | | | | | | | | | | | | | |
| Protestant | | | | | | | 0.852 | (0.362-2.002) | 0.696 | 0.899 | (0.367-2.207) | 0.780 | 0.876 |
| Roman Catholic | | | | | | | 0.596 | (0.195-1.818) | 0.261 | 0.534 | (0.164-1.741) | 0.218 | 0.323 |
| Jewish | | | | | | | 0.000 | (0-100) | 0.983 | 0.000 | (0-100) | 0.983 | 0.964 |
| Other | | | | | | | 1.511 | (0.385-5.931) | 0.529 | 1.525 | (0.368-6.316) | 0.491 | 0.940 |
| McFadden R2 adj | -0.020/-0.004+ | | | -0.025/ -0.009+ | | | 0.045 | | | 0.019 | | | |
| N | 1539 | | | 1539 | | | 1532 | | | 1532 | | | |

Notes: In model 1,2 and 4 we control for the first 20 genetic principal components (PCs). + R2 for model with and without PCs. Abbreviations: OR, odds ratio; CI: confidence interval; p, p-value; PGS, polygenic risk score; NEB, number of children ever born, AFB; age at first birth; PCOS, polycystic ovary syndrome. The difference with the white sample is estimated by testing interactions with ethnicity in the full sample.

**Table SM 9**

Logistic regression results on childlessness in the black male HRS sample

| | Model 1 | | | Model 2 | | | Model 3 | | | Model 4 | | | Diff white sample |
|---|---|---|---|---|---|---|---|---|---|---|---|---|---|
| | OR | 95%CI | p | OR | 95%CI | p | OR | 95%CI | p | OR | 95%CI | p | p |
| Intercept | 0.023 | (0.004-0.143) | 0.000 | 0.022 | (0.003-0.146) | 0.000 | 0.047 | (0.011-0.211) | 0.000 | 0.014 | (0.001-0.178) | 0.000 | |
| PGS (standardized) | | | | | | | | | | | | | |
| NEB | 1.117 | (0.702-1.778) | 0.632 | | | | | | | 1.033 | (0.613-1.742) | 0.895 | 0.715 |
| AFB | 1.108 | (0.766-1.603) | 0.568 | | | | | | | 0.954 | (0.629-1.446) | 0.844 | 0.430 |
| TDS | | | | 0.816 | (0.495-1.343) | 0.414 | | | | 0.803 | (0.466-1.384) | 0.362 | 0.895 |
| Azoospermia | | | | 1.198 | (0.941-1.525) | 0.152 | | | | 1.244 | (0.958-1.615) | 0.089 | 0.232 |
| TGCC | | | | 1.056 | (0.594-1.876) | 0.841 | | | | 1.045 | (0.567-1.927) | 0.864 | 0.958 |
| Infertility | | | | 1.813 | (0.919-3.574) | 0.095 | | | | 2.005 | (0.958-4.194) | 0.065 | 0.645 |
| Hypospadias | | | | 1.015 | (0.712-1.450) | 0.858 | | | | 1.150 | (0.783-1.693) | 0.823 | 0.984 |
| Cryptorchidism | | | | 0.942 | (0.565-1.572) | 0.933 | | | | 0.915 | (0.516-1.624) | 0.476 | 0.932 |
| Socio-demographic | | | | | | | | | | | | | |
| Birth year (standardized) | | | | | | | 1.013 | (0.767-1.338) | 0.878 | 1.009 | (0.753-1.352) | 0.890 | 0.000 |
| Education years (standardized) | | | | | | | 1.196 | (0.871-1.642) | 0.305 | 1.157 | (0.833-1.607) | 0.427 | 0.621 |

**Table SM 9**
Continued

| | Model 1 | | | Model 2 | | | Model 3 | | | Model 4 | | | Diff white sample |
|---|---|---|---|---|---|---|---|---|---|---|---|---|---|
| | OR | 95%CI | p | OR | 95%CI | p | OR | 95%CI | p | OR | 95%CI | p | p |
| Age first marriage (reference is before 21) | | | | | | | | | | | | | |
| 21-25 | | | | | | | 1.635 | (0.512-5.221) | 0.391 | 1.629 | (0.496-5.347) | 0.435 | 0.810 |
| 26-30 | | | | | | | 1.575 | (0.443-5.597) | 0.491 | 1.844 | (0.505-6.74) | 0.356 | 0.272 |
| 31-35 | | | | | | | 2.298 | (0.591-8.941) | 0.197 | 2.491 | (0.62-10.004) | 0.161 | 0.137 |
| 36-40 | | | | | | | 3.741 | (0.951-14.725) | 0.061 | 4.222 | (1.03-17.299) | 0.042 | 0.158 |
| 41+ | | | | | | | 4.88 | (1.453-16.394) | 0.011 | 5.513 | (1.583-19.191) | 0.007 | 0.466 |
| never married | | | | | | | 18.207 | (6.009-55.167) | 0.000 | 21.608 | (6.892-67.74) | 0.000 | 0.000 |
| unknown age | | | | | | | 2.274 | (0.671-7.705) | 0.178 | 2.31 | (0.667-7.993) | 0.176 | 0.046 |
| First occupation (reference are clerks) | | | | | | | | | | | | | |
| army | | | | | | | 2.332 | (0.447-12.175) | 0.312 | 2.046 | (0.393-10.648) | 0.354 | 0.463 |
| farming | | | | | | | 0.778 | (0.122-4.962) | 0.789 | 0.62 | (0.092-4.193) | 0.625 | 0.489 |
| manager | | | | | | | 0.924 | (0.222-3.848) | 0.938 | 0.889 | (0.206-3.83) | 0.930 | 0.940 |
| mechanics, production | | | | | | | 1.144 | (0.377-3.465) | 0.794 | 1.058 | (0.343-3.271) | 0.893 | 0.853 |
| operators | | | | | | | 1.233 | (0.407-3.735) | 0.708 | 1.11 | (0.362-3.406) | 0.825 | 0.841 |
| professionals | | | | | | | 0.992 | (0.241-4.081) | 0.980 | 0.934 | (0.223-3.91) | 0.990 | 0.435 |
| sales | | | | | | | 0.684 | (0.139-3.356) | 0.656 | 0.592 | (0.11-3.171) | 0.570 | 0.646 |
| services | | | | | | | 0.722 | (0.204-2.552) | 0.639 | 0.634 | (0.175-2.298) | 0.530 | 0.550 |

**Table SM 9**

Continued

| Religion (reference is not religious) | | | | | | | | | | | | | |
|---|---|---|---|---|---|---|---|---|---|---|---|---|---|
| | Model 1 | | | Model 2 | | | Model 3 | | | Model 4 | | | Diff white sample |
| | OR | 95%CI | p | OR | 95%CI | p | OR | 95%CI | p | OR | 95%CI | p | p |
| Protestant | | | | | | | 0.633 | (0.295-1.359) | 0.213 | 0.54 | (0.24-1.211) | 0.119 | 0.363 |
| Roman Catholic | | | | | | | 0.542 | (0.175-1.678) | 0.330 | 0.52 | (0.156-1.73) | 0.247 | 0.284 |
| Jewish | | | | | | | 0.000 | (0-100) | 0.985 | 0.000 | (0-100) | 0.983 | 0.966 |
| Other | | | | | | | 0.563 | (0.096-3.306) | 0.530 | 0.595 | (0.086-4.13) | 0.578 | 0.156 |
| McFadden R2 adj | -0.054/-0.014+ | | | -0.059/-0.020+ | | | 0.046 | | | -0.008 | | | |
| N | 901 | | | 901 | | | 896 | | | 896 | | | |

Notes: In model 1, 2 and 4 we control for the first 20 genetic principal components (PCs). + R2 for model with and without PCs.

Abbreviations: OR, odds ratio; CI: confidence interval; p, p-value; PGS, polygenic risk score; NEB, number of children ever born, AFB; age at first birth; PCOS, polycystic ovary syndrome; TDS, testicular dysgenesis syndrome; TGCC, testicular germ cell tumors. The difference with the white sample is estimated by testing interactions with ethnicity in the full sample.

**Table SM 10** Interaction between AFB genetic risk scores and age at marriage in the female HRS sample

| | Model 1: regular controls | | | Model 2: interaction controls | | |
|---|---|---|---|---|---|---|
| | OR | 95% CI | p | OR | 95% CI | p |
| Intercept | 0.007 | (0.001-0.036) | 0.000 | 0.008 | (0-0.184) | 0.073 |
| PGS | | | | | | |
| NEB | 0.879 | (0.749-1.031) | 0.112 | 0.854 | (0.723-1.008) | 0.069 |
| AFB | 0.791 | (0.595-1.052) | 0.095 | 4.654 | (0.405-67.927) | 0.271 |
| PCOS | 1.143 | (0.924-1.413) | 0.259 | 1.163 | (0.935-1.448) | 0.216 |
| Endo | 1.427 | (0.697-2.925) | 0.370 | 1.566 | (0.744-3.299) | 0.248 |
| Mena | 1.067 | (0.955-1.192) | 0.227 | 1.057 | (0.943-1.185) | 0.298 |
| Meno | 1.079 | (0.97-1.199) | 0.159 | 1.073 | (0.961-1.197) | 0.198 |
| Socio-demographic | | | | | | |
| Birth year | 1.352 | (1.213-1.506) | 0.000 | 1.572 | (1.17-2.124) | 0.003 |
| Education years | 1.191 | (1.032-1.376) | 0.015 | 1.197 | (0.819-1.754) | 0.325 |
| Age first marriage (reference is before 21) | | | | | | |
| 21-25 | 1.474 | (1.064-2.043) | 0.015 | 0.187 | (0-577.923) | 0.748 |
| 26-30 | 2.988 | (2.007-4.407) | 0.000 | 0.569 | (0-1820) | 0.870 |
| 31-35 | 5.807 | (3.785-8.809) | 0.000 | 32.12 | (0.004-125000) | 0.408 |
| 36-40 | 2.947 | (1.499-5.354) | 0.001 | 0.582 | (0-35000) | 0.974 |
| 41+ | 4.809 | (3.097-7.33) | 0.000 | 0.000 | (0-17.833) | 0.271 |
| never married | 102.313 | (61.936-175.352) | 0.000 | 2901.27 | (0.166-262000000) | 0.126 |
| unknown age | 3.409 | (2.475-4.718) | 0.000 | 1.322 | (0.005-1440) | 0.859 |
| First occupation (reference are clerks) | | | | | | |
| army | 1.201 | (0.059-8.354) | 0.884 | 0.967 | (0.047-6.967) | 0.985 |
| farming | 1.575 | (0.543-3.845) | 0.379 | 1.356 | (0.464-3.386) | 0.573 |
| manager | 1.43 | (1.04-1.959) | 0.037 | 1.374 | (0.984-1.909) | 0.079 |
| mechanics, production | 1.024 | (0.757-1.385) | 0.932 | 1.022 | (0.748-1.395) | 0.929 |
| operators | 0.563 | (0.327-0.924) | 0.027 | 0.582 | (0.337-0.961) | 0.039 |
| professionals | 1.39 | (1.043-1.856) | 0.039 | 1.353 | (0.998-1.836) | 0.063 |
| sales | 1.03 | (0.714-1.466) | 0.981 | 1.018 | (0.697-1.466) | 0.956 |
| services | 0.738 | (0.512-1.05) | 0.097 | 0.721 | (0.497-1.034) | 0.082 |
| Religion (reference is not religious) | | | | | | |
| Other | 1.207 | (0.491-2.717) | 0.975 | 0.612 | (0.03-4.093) | 0.490 |
| Protestant | 0.973 | (0.696-1.38) | 0.962 | 0.677 | (0.326-1.558) | 0.248 |
| Roman Catholic | 0.971 | (0.673-1.415) | 0.573 | 0.494 | (0.196-1.29) | 0.745 |
| Jewish | 0.702 | (0.209-2.289) | 0.604 | 0.57 | (0.015-10.669) | 0.733 |

**Table SM 10** Continued

| | Model 1: regular controls | | | Model 2: interaction controls | | |
|---|---|---|---|---|---|---|
| | OR | 95% CI | p | OR | 95% CI | p |
| Interaction PGS AFB * Age marriage (reference is before 21) | | | | | | |
| 21-25 | 1.119 | (0.772-1.622) | 0.526 | 1.217 | (0.812-1.825) | 0.347 |
| 26-30 | 1.276 | (0.849-1.92) | 0.245 | 1.548 | (0.973-2.469) | 0.082 |
| 31-35 | 1.492 | (0.929-2.406) | 0.084 | 1.766 | (1.036-3.041) | 0.035 |
| 36-40 | 2.732 | (1.512-5.209) | 0.001 | 3.471 | (1.669-7.634) | 0.001 |
| 41+ | 1.763 | (1.111-2.824) | 0.028 | 1.534 | (0.898-2.628) | 0.164 |
| never married | 1.934 | (1.074-3.563) | 0.028 | 1.633 | (0.763-3.52) | 0.210 |
| unknown age | 1.233 | (0.875-1.74) | 0.211 | 1.194 | (0.801-1.781) | 0.401 |
| Interactions birth year * marriage (reference is before 21) | | | | | | |
| 21-25 | | | | 0.84 | (0.572-1.229) | 0.353 |
| 26-30 | | | | 1.316 | (0.828-2.111) | 0.261 |
| 31-35 | | | | 0.719 | (0.428-1.206) | 0.221 |
| 36-40 | | | | 1.119 | (0.516-2.448) | 0.787 |
| 41+ | | | | 3.025 | (1.468-6.518) | 0.001 |
| never married | | | | 0.235 | (0.106-0.476) | 0.000 |
| unknown age | | | | 0.906 | (0.637-1.284) | 0.582 |
| Interactions years of education * marriage (reference is before 21) | | | | | | |
| 21-25 | | | | 1.011 | (0.628-1.633) | 0.999 |
| 26-30 | | | | 0.934 | (0.543-1.623) | 0.724 |
| 31-35 | | | | 1.21 | (0.666-2.218) | 0.580 |
| 36-40 | | | | 0.774 | (0.357-1.691) | 0.518 |
| 41+ | | | | 1.477 | (0.804-2.747) | 0.318 |
| never married | | | | 1.89 | (0.952-3.947) | 0.083 |
| unknown age | | | | 0.778 | (0.503-1.201) | 0.276 |
| Interactions religion (ref is not religious) * marriage (reference is before 21) | | | | | | |
| 21-25 * protestant | | | | 1.01 | (0.351-2.841) | 0.851 |
| 26-30 * protestant | | | | 1.505 | (0.483-4.599) | 0.628 |
| 31-35 * protestant | | | | 2.281 | (0.633-8.203) | 0.269 |
| 36-40 * protestant | | | | 2.162 | (0.301-22.819) | 0.541 |
| 41+ * protestant | | | | 2.682 | (0.476-16.919) | 0.354 |
| never married * protestant | | | | 6.462 | (1.171-35.742) | 0.043 |
| unknown age * protestant | | | | 2.601 | (0.81-8.875) | 0.162 |
| 21-25 * catholic | | | | 1.3 | (0.393-4.291) | 0.830 |
| 26-30 * catholic | | | | 1.614 | (0.442-5.843) | 0.577 |
| 31-35 * catholic | | | | 2.362 | (0.576-9.665) | 0.285 |
| 36-40 * catholic | | | | 5.311 | (0.59-64.044) | 0.191 |
| 41+ * catholic | | | | 5.321 | (0.808-38.556) | 0.119 |
| never married * catholic | | | | 14.994 | (2.076-118.41) | 0.012 |
| unknown age * catholic | | | | 2.942 | (0.8-11.498) | 0.157 |

**Table SM 10** Continued

| | Model 1: regular controls | | | Model 2: interaction controls | | |
|---|---|---|---|---|---|---|
| | OR | 95% CI | p | OR | 95% CI | p |
| 21-25 * jewish | | | | 1.483 | (0.044-70.823) | 0.858 |
| 26-30 * jewish | | | | 0.874 | (0.027-41.019) | 0.926 |
| 31-35 * jewish | | | | 0.000 | (0-Inf) | 0.986 |
| 36-40 * jewish | | | | 22.696 | (0.205-4340.408) | 0.215 |
| 41+ * jewish | | | | 0.000 | (0-Inf) | 0.994 |
| never married * jewish | | | | Inf | (0-Inf) | 0.978 |
| unknown age * jewish | | | | 3.88 | (0.159-154.571) | 0.456 |
| 21-25 * other | | | | 1.208 | (0.035-43.334) | 0.963 |
| 26-30 * other | | | | 0.000 | (0-Inf) | 0.988 |
| 31-35 * other | | | | 6.543 | (0.118-467.12) | 0.377 |
| 36-40 * other | | | | 0.000 | (0-Inf) | 0.993 |
| 41+ * other | | | | Inf | (0-Inf) | 0.986 |
| never married * other | | | | Inf | (0-Inf) | 0.973 |
| unknown age * other | | | | 4.79 | (0.273-147.428) | 0.316 |
| Interactions pc1 * marriage (reference is before 21) | | | | | | |
| 21-25 | | | | Inf | (0-Inf) | 0.392 |
| 26-30 | | | | Inf | (0-Inf) | 0.470 |
| 31-35 | | | | 0.000 | (0-Inf) | 0.710 |
| 36-40 | | | | Inf | (0-Inf) | 0.758 |
| 41+ | | | | Inf. | (0-Inf) | 0.137 |
| never married | | | | 0.000 | (0-Inf) | 0.742 |
| unknown age | | | | Inf | (0-Inf) | 0.903 |
| Interactions pc2 * marriage (reference is before 21) | | | | | | |
| 21-25 | | | | 0.000 | (0-Inf) | 0.189 |
| 26-30 | | | | 0.000 | (0-Inf) | 0.108 |
| 31-35 | | | | 0.000 | (0-Inf) | 0.655 |
| 36-40 | | | | 0.000 | (0-Inf) | 0.216 |
| 41+ | | | | 0.000 | (0-Inf) | 0.160 |
| never married | | | | 0.000 | (0-Inf) | 0.225 |
| unknown age | | | | 0.000 | (0-Inf) | 0.736 |
| Interactions pc3 * marriage (reference is before 21) | | | | | | |
| 21-25 | | | | 0.000 | (0-Inf) | 0.757 |
| 26-30 | | | | 0.29 | (0-Inf) | 0.950 |
| 31-35 | | | | 0.000 | (0-Inf) | 0.121 |
| 36-40 | | | | 0.000 | (0-Inf) | 0.837 |
| 41+ | | | | 0.000 | (0-Inf) | 0.134 |
| never married | | | | Inf. | (0-Inf) | 0.085 |
| unknown age | | | | Inf. | (0-Inf) | 0.871 |

**Table SM 10** Continued

| | Model 1: regular controls | | | Model 2: interaction controls | | |
|---|---|---|---|---|---|---|
| | OR | 95% CI | p | OR | 95% CI | p |
| Interactions pc4 * marriage (reference is before 21) | | | | | | |
| 21-25 | | | | Inf. | (0-Inf) | 0.683 |
| 26-30 | | | | 0.14 | (0-Inf) | 0.951 |
| 31-35 | | | | 0.000 | (0-Inf) | 0.579 |
| 36-40 | | | | Inf. | (0-Inf) | 0.498 |
| 41+ | | | | 0.000 | (0-0) | 0.062 |
| never married | | | | 0.000 | (0-Inf) | 0.887 |
| unknown age | | | | 0.008 | (0-Inf) | 0.976 |
| Interactions pc5 * marriage (reference is before 21) | | | | | | |
| 21-25 | | | | 0.000 | (0-Inf) | 0.203 |
| 26-30 | | | | 0.000 | (0-Inf) | 0.450 |
| 31-35 | | | | 14366.000 | (0-Inf) | 0.741 |
| 36-40 | | | | 0.000 | (0-Inf) | 0.576 |
| 41+ | | | | 0.000 | (0-Inf) | 0.820 |
| never married | | | | 18.96 | (0-Inf) | 0.854 |
| unknown age | | | | 0.000 | (0-Inf) | 0.424 |
| Interaction birth year * AFB PGS | | | | 0.913 | (0.8-1.042) | 0.175 |
| Interaction education years * AFB PGS | | | | 0.961 | (0.821-1.122) | 0.573 |
| Interaction religion (ref is not religious) * AFB PGS | | | | | | |
| Protestant | | | | 0.692 | (0.455-1.043) | 0.093 |
| Roman Catholic | | | | 0.822 | (0.52-1.294) | 0.415 |
| Jewish | | | | 0.856 | (0.293-2.634) | 0.748 |
| Other | | | | 0.274 | (0.04-1.453) | 0.152 |
| Interaction pc1 * AFB PGS | | | | 0.000 | (0-Inf) | 0.299 |
| Interaction pc2 * AFB PGS | | | | 121.821 | (0-Inf) | 0.986 |
| Interaction pc3 * AFB PGS | | | | 0.000 | (0-Inf) | 0.425 |
| Interaction pc4 * AFB PGS | | | | Inf. | (0-Inf) | 0.045 |
| Interaction pc5 * AFB PGS | | | | 0.055 | (0-124000) | 0.749 |
| McFadden R2adj | 0.179 | | | 0.163 | | |
| N | 6133 | | | 6133 | | |

Notes: We control for the first 20 genetic principal components (PCs). Abbreviations: OR, odds ratio; CI: confidence interval; p, p-value; PGS, polygenic risk score; NEB, number of children ever born, AFB; age at first birth; PCOS, polycystic ovary syndrome. Due to perfect separation the estimates for the interaction between some of the religious categories and age at marriage, and the principal components with age at marriage, the effects of these interactions could not be estimated.

**Table SM 11** The relationship between AFB genetic risk scores and age at marriage and childlessness in the female HRS sample, descriptively

| | | Age at first marriage | | | | | | | | |
|---|---|---|---|---|---|---|---|---|---|---|
| | | <21 | 21-25 | 26-30 | 31-35 | 36-40 | 41+ | never married | unknown age | All |
| N | | 1879 | 1526 | 450 | 234 | 146 | 280 | 178 | 1476 | 6169 |
| N | with child | 1801 | 1413 | 381 | 175 | 119 | 227 | 26 | 1326 | 5468 |
| N | childless | 78 | 111 | 69 | 58 | 27 | 53 | 152 | 150 | 698 |
| % | with child | 0.958 | 0.927 | 0.847 | 0.751 | 0.815 | 0.811 | 0.146 | 0.898 | 0.887 |
| % | childless | 0.042 | 0.073 | 0.153 | 0.249 | 0.185 | 0.189 | 0.854 | 0.102 | 0.113 |
| Mean AFB PGS | with child | 0.176 | 0.443 | 0.461 | 0.272 | 0.207 | 0.186 | 0.206 | 0.387 | 0.320 |
| Mean AFB PGS | childless | 0.080 | 0.374 | 0.551 | 0.480 | 0.901 | 0.566 | 0.530 | 0.393 | 0.440 |

Abbreviations: PGS, polygenic risk score; AFB, age at first birth; NEB, number of children ever born

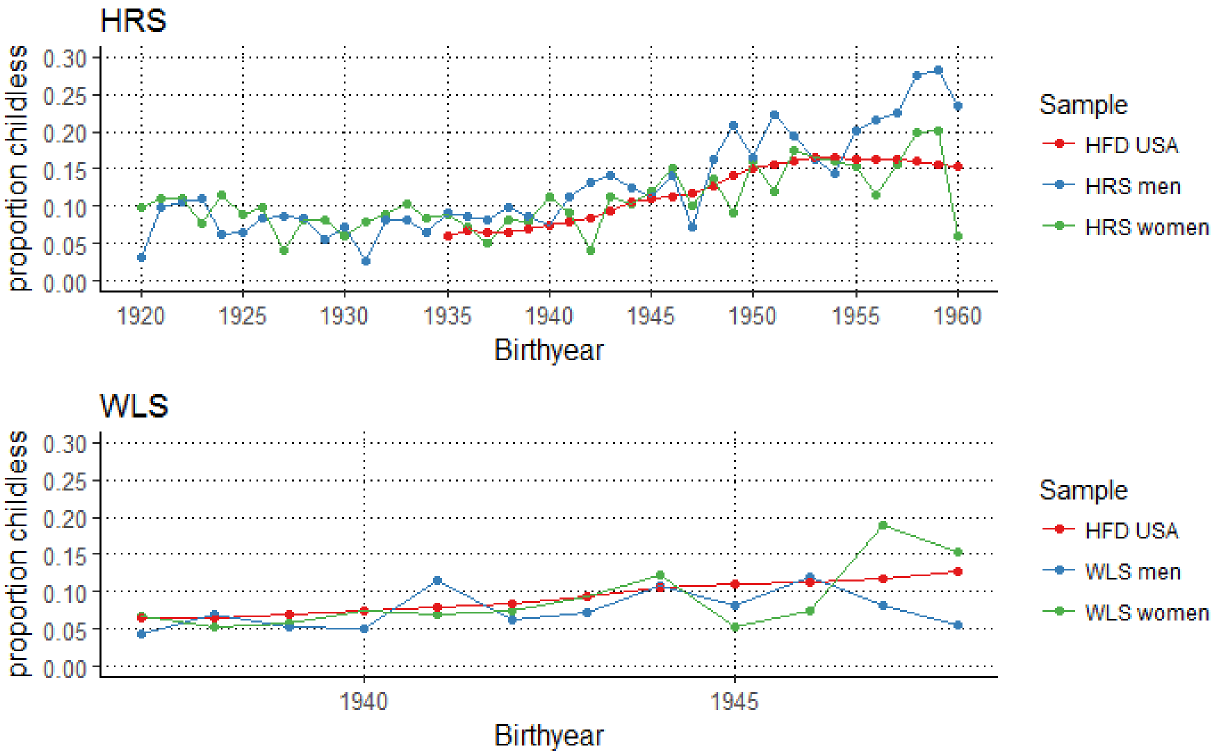


**Figure SM1** Childlessness levels in our data and as reported in the human fertility database (HFD) for the USA. For the HRS we show the estimates for the white sample.

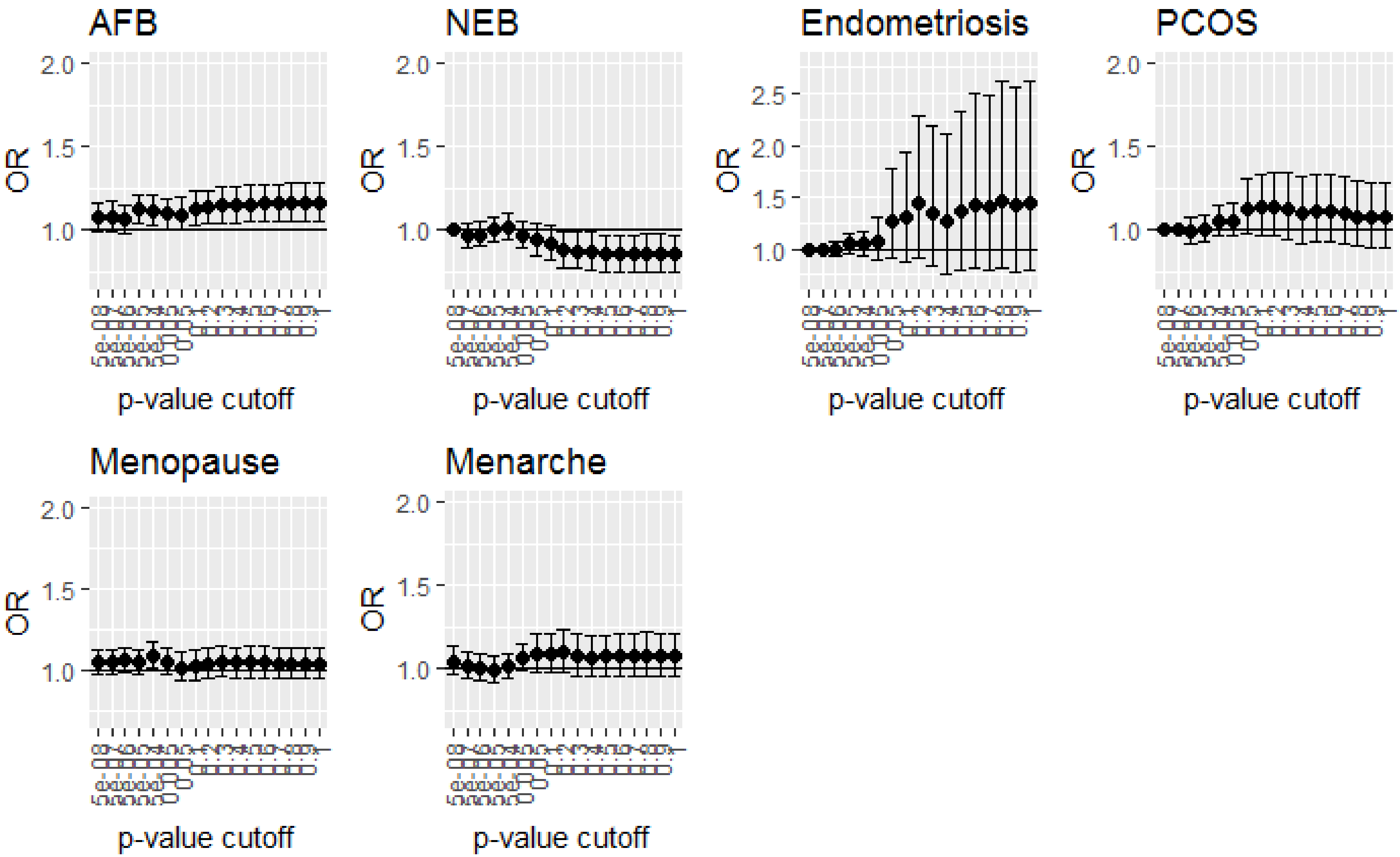


**Figure SM2** PGS effects on childlessness using different p-value cutoffsfor women in the HRS sample, univariate models with 20 principal components as control variables included. OR and 95% CI displayed. Abbreviations: OR, odds ratio; AFB, age at first birth; NEB, number of children ever born; PCOS, polycystic ovary syndrome

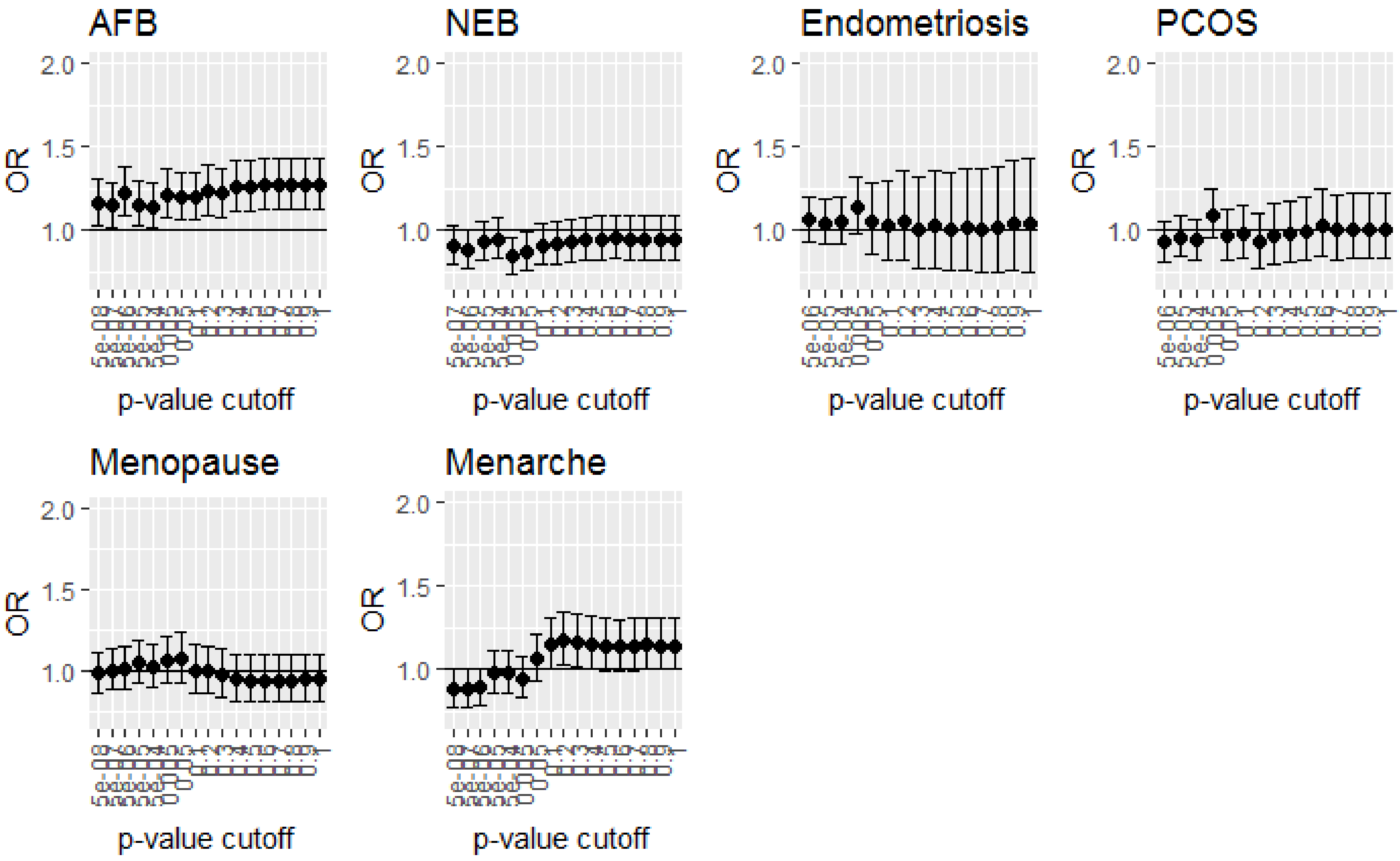


**Figure SM3** PGS effects on childlessness using different p-value cutoffs for women in the WLS sample, univariate models with 20 principal components as control variables included. OR and 95% CI displayed. Abbreviations: OR, odds ratio; AFB, age at first birth; NEB, number of children ever born; PCOS, polycystic ovary syndrome

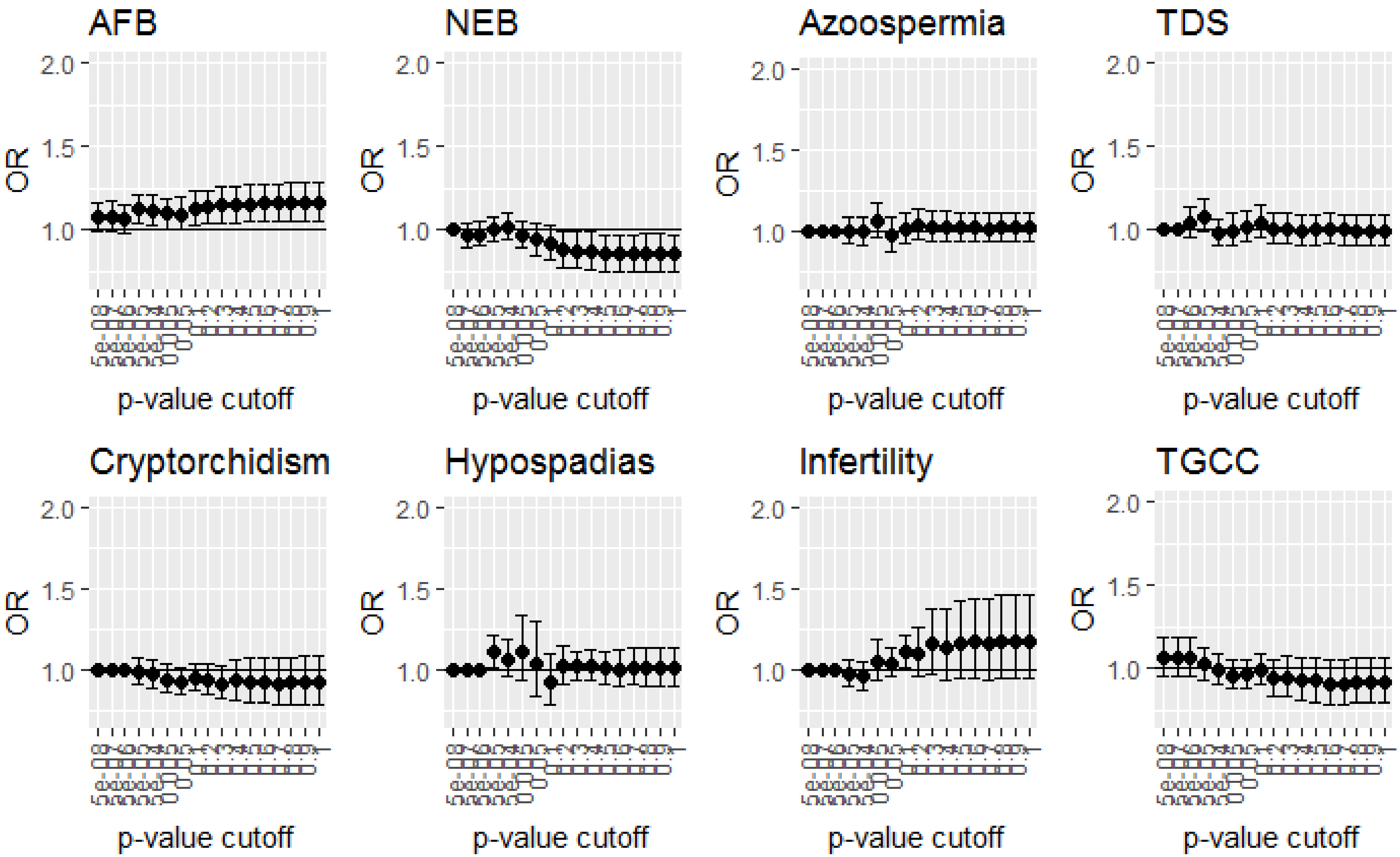


**Figure SM4** PGS effects on childlessness using different p-value cutoffs for men in the HRS sample, univariate models with 20 principal components as control variables included. OR and 95% CI displayed. Abbreviations: OR, odds ratio; AFB, age at first birth; NEB, number of children ever born; TDS, testicular dysgenesis syndrome; TGCC, testicular germ cell tumors.

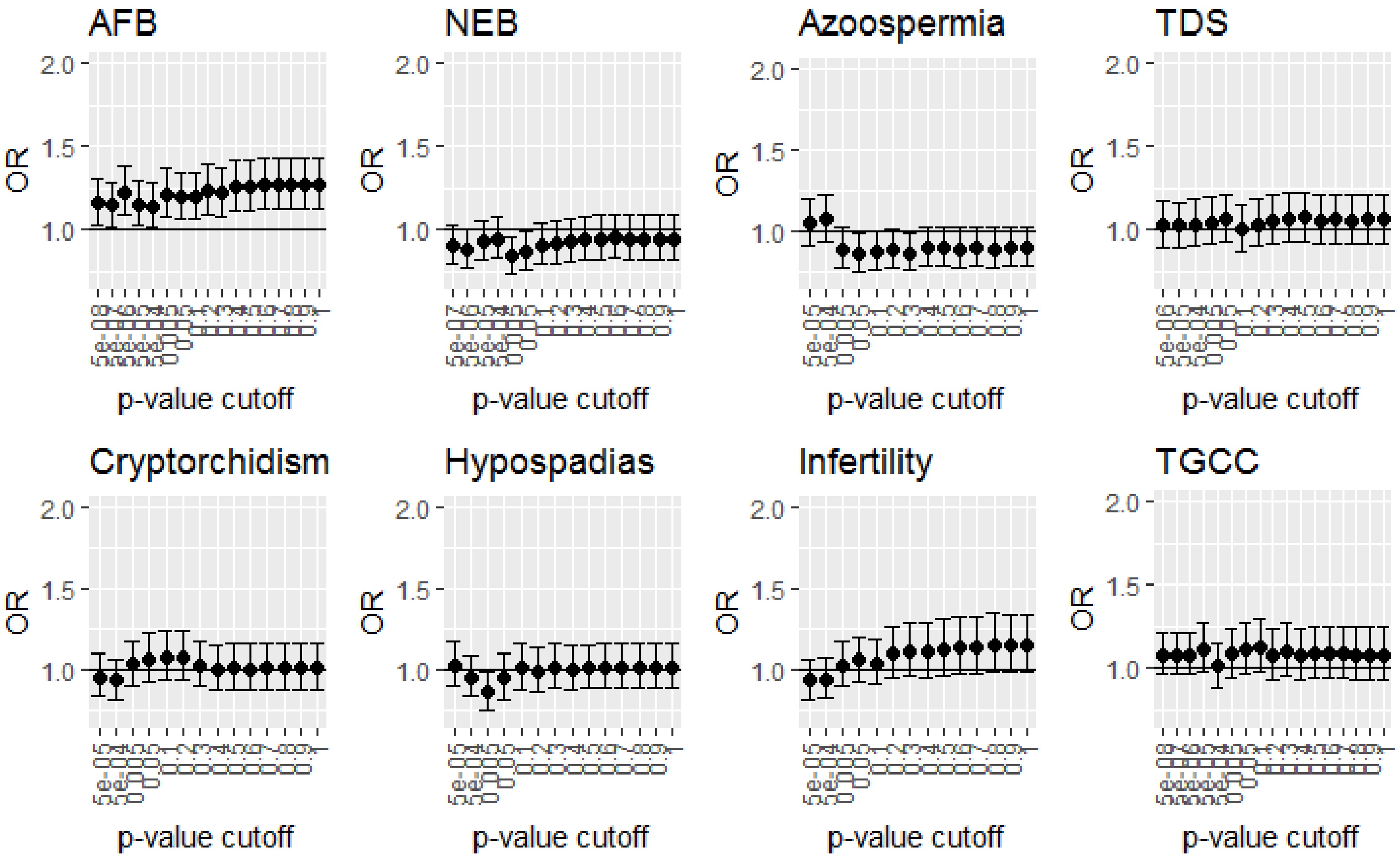


**Figure SM5** PGS effects on childlessness using different p-value cutoffs for women in the HRS sample, univariate models with 20 principal components as control variables included. OR and 95% CI displayed. Abbreviations: OR, odds ratio; AFB, age at first birth; NEB, number of children ever born; TDS, testicular dysgenesis syndrome; TGCC, testicular germ cell tumors.

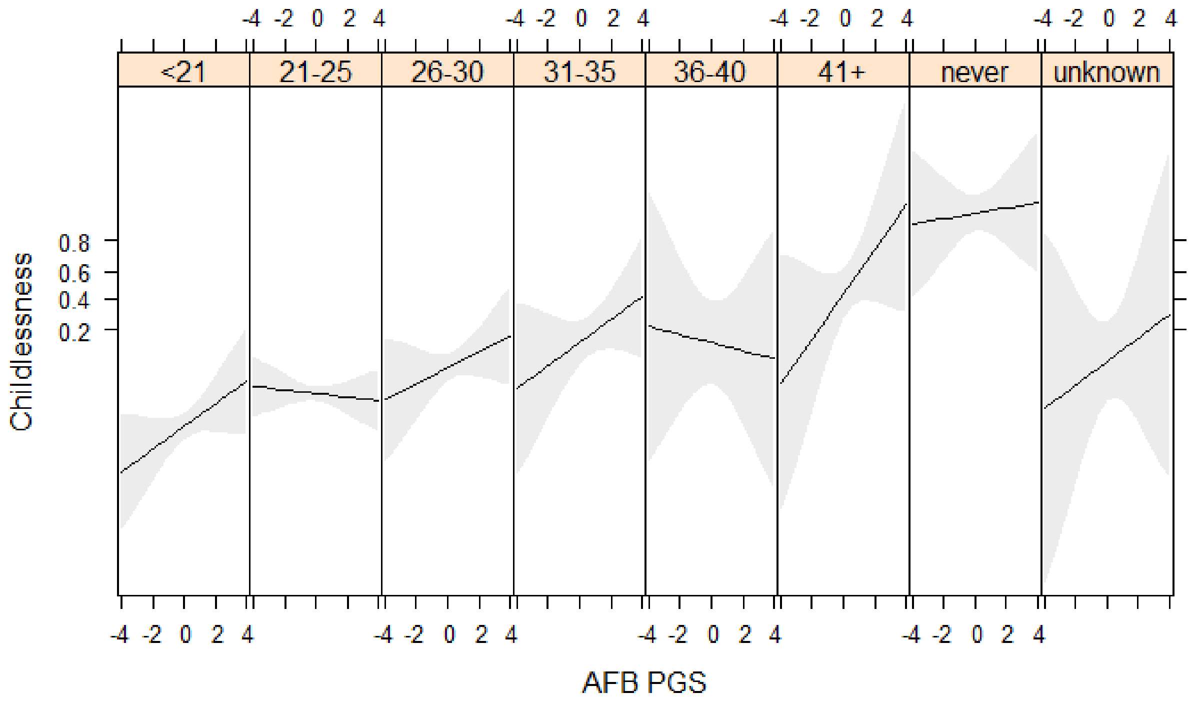


**Figure SM6** AFB genes and their interaction with age at marriage in the female WLS sample
